# COVID-19 Immunologic Antiviral Therapy With Omalizumab (CIAO)—a Randomized Controlled Clinical Trial

**DOI:** 10.1093/ofid/ofae102

**Published:** 2024-02-23

**Authors:** Michelle Le, Lauren Khoury, Yang Lu, Connor Prosty, Maxime Cormier, Mathew P Cheng, Robert Fowler, Srinivas Murthy, Jennifer L Y Tsang, Moshe Ben-Shoshan, Elham Rahme, Shirin Golchi, Nandini Dendukuri, Todd C Lee, Elena Netchiporouk

**Affiliations:** Division of Dermatology, Department of Medicine, McGill University, Montreal, QC, Canada; Faculty of Medicine, McGill University, Montreal, QC, Canada; Department of Epidemiology, Biostatistics, and Occupational Health, McGill University, Montreal, QC, Canada; Faculty of Medicine, McGill University, Montreal, QC, Canada; Division of Respiratory Medicine, Department of Medicine, McGill University, Montreal, QC, Canada; Divisions of Infectious Diseases & Medical Microbiology, McGill University, McGill's Interdisciplinary Initiative in Infection and Immunity, Montreal, QC, Canada; Department of Critical Care Medicine, Sunnybrook Health Sciences Centre, Toronto, ON, Canada; Department of Pediatrics, Faculty of Medicine, University of British Columbia, Vancouver, BC, Canada; Niagara Health Knowledge Institute, Niagara Health, St. Catharines, ON, Canada; Division of Allergy, Immunology and Dermatology, Department of Pediatrics, McGill University, Montreal, QC, Canada; Department of Epidemiology, Biostatistics, and Occupational Health, McGill University, Montreal, QC, Canada; Department of Epidemiology, Biostatistics, and Occupational Health, McGill University, Montreal, QC, Canada; Department of Epidemiology, Biostatistics, and Occupational Health, McGill University, Montreal, QC, Canada; Divisions of Infectious Diseases & Medical Microbiology, McGill University, McGill's Interdisciplinary Initiative in Infection and Immunity, Montreal, QC, Canada; Division of Dermatology, Department of Medicine, McGill University, Montreal, QC, Canada

**Keywords:** acute respiratory distress syndrome, coronavirus, COVID-19, omalizumab, SARS-CoV2

## Abstract

**Background:**

Omalizumab is an anti-immunoglobulin E monoclonal antibody used to treat moderate to severe chronic idiopathic urticaria, asthma, and nasal polyps. Recent research suggested that omalizumab may enhance the innate antiviral response and have anti-inflammatory properties.

**Objective:**

We aimed to investigate the efficacy and safety of omalizumab in adults hospitalized for coronavirus disease 2019 (COVID-19) pneumonia.

**Methods:**

This was a phase II randomized, double blind, placebo-controlled trial comparing omalizumab with placebo (in addition to standard of care) in hospitalized patients with COVID-19. The primary endpoint was the composite of mechanical ventilation and/or death at day 14. Secondary endpoints included all-cause mortality at day 28, time to clinical improvement, and duration of hospitalization.

**Results:**

Of 41 patients recruited, 40 were randomized (20 received the study drug and 20 placebo). The median age of the patients was 74 years and 55.0% were male. Omalizumab was associated with a 92.6% posterior probability of a reduction in mechanical ventilation and death on day 14 with an adjusted odds ratio of 0.11 (95% credible interval 0.002-2.05). Omalizumab was also associated with a 75.9% posterior probability of reduced all-cause mortality on day 28 with an adjusted odds ratio of 0.49 (95% credible interval, 0.06-3.90). No statistically significant differences were found for the time to clinical improvement and duration of hospitalization. Numerically fewer adverse events were reported in the omalizumab group and there were no drug-related serious adverse events.

**Conclusions:**

These results suggest that omalizumab could prove protective against death and mechanical ventilation in hospitalized patients with COVID-19. This study could also support the development of a phase III trial program investigating the antiviral and anti-inflammatory effect of omalizumab for severe respiratory viral illnesses requiring hospital admission.

ClinicalTrials.gov ID: NCT04720612

## Teaser

In this, double-blind multicenter randomized phase II clinical trial, omalizumab had a 93% posterior probability at reducing COVID-19 mortality and/or mechanical ventilation at day 14, and a 75% probability at reducing all-cause mortality at day 28 regardless of the presence of atopy. Its use was safe in severely ill patients. This study supports the development of a phase III program to study the antiviral and anti-inflammatory effect of omalizumab in severe respiratory viral illnesses.

The coronavirus disease 2019 (COVID-19) pandemic, caused by the severe acute respiratory syndrome coronavirus 2 (SARS-CoV-2) virus, has caused unimaginable morbidity and mortality worldwide. SARS-CoV-2 infection follows 2 phases: an acute phase, characterized by viral replication; and a later phase, driven by hyperinflammation [1, 2]. The acute respiratory distress syndrome arising from hyperinflammation is 1 of the main drivers of attributable mortality in patients with COVID-19 [1, 2]. Despite incredible innovations in vaccine development, mass vaccination campaigns (including boosters), and resolved natural infections, waning of protection occurs [3, 4]. In addition, with the emergence of SARS-CoV-2 variants, COVID-19 will not be eradicated and endemic phase is predicted if not already established [5–7] Broad spectrum anti-inflammatory agents, notably dexamethasone, Janus kinase inhibitors and interleukin-6 (IL-6) inhibitors (eg, tocilizumab) reduce mortality in hospitalized patients with severe COVID-19 disease and are commonly used in clinical practice [1]. Nonetheless, these current agents are not universally effective and may be contraindicated or associated with significant side effects, especially in vulnerable populations (eg, pregnant, immunosuppressed, elderly). Additional research can also better prepare us for other potential future epidemics or pandemics.

Mast cells are immune cells strategically placed in barrier tissues (eg, respiratory tract, nasal cavity) that have both innate and adaptive immunity effector and regulatory functions [8]. Mast cells are activated during respiratory viral infections through a variety of local signals (from the virus or the host) culminating in degranulation with cytokine release (eg, IL-1, IL-6, tumor necrosis factor-α, interferon), complement/coagulation activation, and regulation of T-cell responses [9–11]. Serum mast cell mediators correlated with disease severity in viral infections such as influenza, dengue, and COVID-19, whereas mast cell hyperplasia was hypothesized to be associated with prolonged symptoms found in long COVID and postviral fatigue syndromes [12, 13].

Omalizumab is a humanized anti-immunoglobulin E (IgE) monoclonal antibody (mAb) approved for the treatment of several mast cell–mediated diseases, notably moderate-severe allergic asthma, chronic idiopathic urticaria, and nasal polyps [14]. The drug has an excellent safety profile with more than 1.99 million patient-years of exposure including children, pregnant patients, the elderly, and individuals with multiple comorbidities [15, 16]. Mild adverse drug reactions (AEs) (ie, malaise, urticaria, dizziness, skin rash) occur in ∼8% of patients, and the rate of serious adverse events (SAEs) is 1.2%, a similar rate to placebo [17–19]. The incidence of the most important SAE, anaphylaxis, is 0.2% and usually occurs in patients with a history of anaphylaxis [20, 21].

Omalizumab binds the free serum IgE and prevents it from binding the IgE receptors (FcɛRI and FcɛRII or CD23) on mast cells, antigen-presenting cells, and other leukocytes [22–24]. Omalizumab also reduces mast cells degranulation and downstream mediator release [22]. In vitro and in vivo data highlighted that omalizumab may also have antiviral properties [34]. Notably, a single dose of omalizumab decreased inflammation (eg, IL-1β, IL-6, tumor necrosis factor-α) in bronchial epithelial cell-line model and prevented death from lipopolysaccharide-induced acute lung injury in mice [25, 26]. Observational studies and randomized controlled trials (RCTs) of patients with asthma showed that omalizumab decreased the incidence, duration, and severity of rhinovirus infections [27, 28].

On the basis of its mechanism of action, experimental evidence, and the observational data in asthma, we aimed to conduct a phase II double blind RCT to investigate the potential efficacy of omalizumab in adult patients hospitalized for COVID-19 pneumonia.

## METHODS

This RCT is reported in accordance with the CONSORT reporting guidelines [29].

### Ethics

This trial was approved by Health Canada, the McGill University Health Center (MUHC) Research Institute, and Clinical Trials Ontario ethics review boards, was registered at www.clinicaltrials.gov (NCT04720612), and was conducted following the tenets of Good Clinical Practice. Informed consent was obtained from all patients and/or their legal representatives. The Health Canada–approved protocol with its statistical analysis plan is available in the Appendix.

### Trial Design and Oversight

This was a phase II double blind multicenter, placebo-controlled RCT of a single dose of omalizumab in addition to standard of care treatment among hospitalized patients with COVID-19 that was conducted between December 2021 and November 2022. The COVID-19 Immunologic Antiviral Therapy With Omalizumab was a substudy of the Pan-Canadian Clinical Trial Consortium—the Canadian Treatments for COVID-19. Three hospitals across 2 Canadian provinces participated: MUHC in Montreal, Quebec; Sunnybrook Health Sciences Centre in Toronto, Ontario; and Niagara Health, in Niagara, Ontario. The Research Institute of the MUHC was the sponsor of the trial. The study was funded by the Ministère de l’Économie et Innovation/Fonds de Recherche Santé Québec and the MUHC Foundation. Novartis pharmaceuticals donated the omalizumab for the study but had no role in data analysis or manuscript preparation.

Adult patients hospitalized for COVID-19 pneumonia were eligible if they had polymerase chain reaction–confirmed SARS-CoV-2 infection and met either of the respiratory criteria: dyspnea at rest or during minimal activity; respiratory rate > 22/min; and partial pressure of oxygen in arterial blood < 65 mm Hg or oxygen saturation < 90% or new or worsening infiltrate on chest radiography. Patients with known hypersensitivity to omalizumab or its excipients, inability to give consent themselves or via a proxy, patients who received omalizumab or another anti-IgE molecule in the past 12 months, or patients who received another cytokine/receptor mAb before study initiation were excluded. As the COVID-19 pandemic evolved, a protocol amendment was made after study initiation to allow patients receiving antiviral therapies as well as patients receiving mAbs that did not act on cytokines or their receptors (eg, denosumab) to participate in the trial.

### Dose Justification

Because the safety signal in omalizumab studies was not dose dependent, but preclinical and clinical data suggested that efficacy is dose dependent [30], we chose to administer the highest approved dose of omalizumab for asthma patients (ie, 375 mg subcutaneous dose injection).

### Randomization and Allocation Concealment

A permuted block randomization (4 patients per block) was performed on REDCap, a web-based system with concealment of the study arm assignment and stratified according to the level of respiratory support (no oxygen support; oxygen support with nasal cannula or mask; noninvasive ventilation; and invasive ventilation or extracorporeal membrane oxygenation). Patients received the current standard of care for hospitalized patients with COVID-19 and were randomized 1:1 to receive either a single dose of 375 mg omalizumab subcutaneously or a placebo.

### Clinical Assessment Procedures

Patient demographics (self-reported), date of admission and symptom onset, relevant medical and medication history, vital status, and physical examination/level of respiratory support were reported at baseline. On days 1, 5, 14, and 28 after randomization, vital status, physical examination/level of respiratory support, and AEs were assessed via chart review and reported through an online follow-up form. Further information regarding coenrollment in other clinical trials, vital signs, and AEs (including dates, cause of death, and severity of AEs) were elicited when necessary. If patients were discharged before day 28, they were contacted by phone to obtain outcomes data. Sera for exploratory analyses (ie, IgE, tryptase, cytokines) were collected on days 0, 2, 7, and 14 in collaboration with the MUHC COVID-19 biobank.

### Outcome Measures

The primary outcome measure was the composite of death and/or need for mechanical ventilation on day 14. The secondary outcomes were all-cause in-hospital mortality on day 28, time to clinical improvement (ie, improvement of 2 points on the 9-category ordinal scale as recommended by the World Health Organization [WHO] R&D Blueprint expert group (Supplementary Table 1)), duration of hospitalization, duration of mechanical ventilation, and safety of omalizumab in hospitalized patients with COVID-19. Although serial serum samples were collected from MUHC participants, results from exploratory analyses will be reported separately.

### Sample Size Calculation and Protocol Changes

We originally planned to conduct an adaptive trial with 2 interim analyses for the primary outcome at 33 and 66 patients per arm and a maximum sample size of 100 patients per arm (200 total patients). The stopping criteria were specified to achieve 80% power, while controlling the overall type I error rate below 5% and accounting for 5% loss to follow-up. If the posterior probability that the odds ratio (OR) > 0.8 exceeded 0.95 or the posterior probability that OR ≤ 0.6 exceeded 0.8, the trial would have been stopped for futility or efficacy, respectively. Otherwise, the trial would have continued until a sample size of 100 patients per treatment arm was reached (Supplementary protocol). In November 2022, because of a decline in hospital admissions for severe COVID-19 and a lack of funding to continue indefinitely to the preplanned sample size, a decision was made (before any data analysis) to terminate the trial.

### Statistical Analysis

Patient demographics and clinical characteristics were summarized using descriptive statistics (median and interquartile range [IQR] for continuous data and percentage for categorical data).

Efficacy analyses were conducted for all randomized patients according to an intention-to-treat perspective. Safety outcomes were analyzed in the as-treated population. We used Bayesian logistic regression to estimate the primary efficacy outcome while a priori adjusting for stratification, age, sex, and vaccination status. Sensitivity analyses without adjustment were conducted. The median adjusted OR (aOR) and 95% credible interval (CrI) were reported for all parameters along with the posterior probability of superiority.

A Bayesian mixed-effects logistic regression model was used to assess secondary outcome measures including all-cause mortality at day 28, the effect of time on clinical improvement, and the duration of hospitalization adjusted for stratification, age, sex, and vaccination status. Kaplan-Meier survival curves were generated to visualize mortality.

For all the models considered, a Markov chain Monte Carlo approach was used to sample from the posterior distributions using the rjags package within the R statistical programming environment (version 4.2.2). We used noninformative normal (mean = 0, variance = 10 000) previous distributions over all parameters. The jags model is provided in the Supplementary methods. Three independent Markov chain Monte Carlo chains of 25 000 iterations were run; the first 5000 iterations of each chain were discarded [31, 32]. This study used a Bayesian statistical framework to analyze the data. Unlike traditional frequentist statistics, Bayesian analysis relies on posterior distributions and credible intervals rather than *P* values. As a result, *P* values were not included in the analysis.

## RESULTS

### Patient Demographics

**Figure 1.**
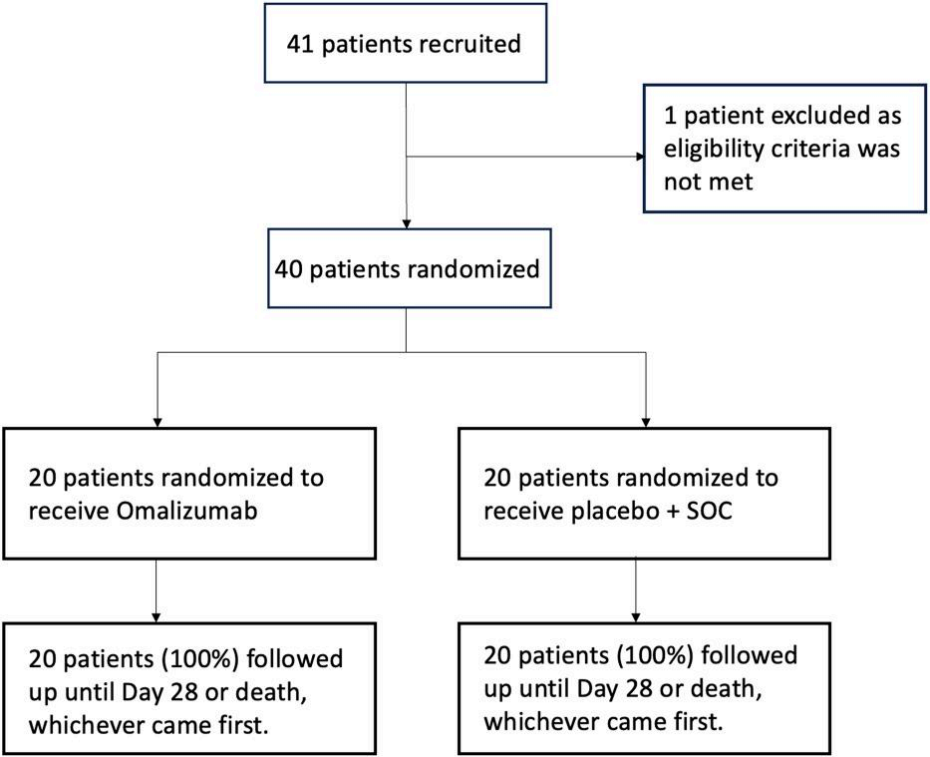
Participant flow diagram.

Of the 41 patients recruited from December 2021 to November 2022, 40 were randomized 1:1 to receive study drug or placebo. One patient was initially recruited who did not meet the eligibility criteria. As such, that patient was not randomized and was excluded from all statistical analyses (Figure 1).

Patient characteristics are presented in Table 1. The groups were well balanced with regard to age, sex, comorbidities, including the Charlson comorbidity index, vaccination status, WHO 9-point ordinal scale, time from hospital admission to enrollment, and cotreatment with dexamethasone. The median age of the patients was 74 years and 55.0% were male. The median time patients underwent randomization was 2 days after hospitalization (IQR, 1, 2) corresponding to a median of 10 days after symptom onset (IQR, 4, 15). The majority of patients received supplemental oxygen by mask/nasal prongs (42.5%) or noninvasive ventilation/high-flow oxygen (27.5%) at randomization. The median Charlson comorbidity index score was 4 (IQR, 2, 5) with the most common medical comorbidities being diabetes (50.0%), cardiovascular disease (42.5%), and chronic kidney disease (25.0%). The majority of the patients had received at least 2 doses of COVID-19 vaccine (71.8%) at the time of enrollment. Most patients received concomitant dexamethasone as part of standard of care (97.5%, n = 39); in addition, 45% (n = 18) of patients received remdesivir, whereas 15.4% (n = 6) of patients received baricitinib and 2.6% (n = 1) of patients received tocilizumab during the study.

**Table 1.** Baseline Patients’ Characteristics

Parameter	Placebo (n = 20)	Omalizumab (n = 20)	Overall (n = 40)
Age—y			
Median (IQR)	72 (60, 79)	74 (59, 82)	74 (60, 82)
Age category—y, no. (%)			
≥65	12 (60.0)	14 (70.0)	26 (65.0)
Sex—no. (%)			
Male	12 (60.0)	10 (50.0)	22 (55.0)
Race—no. (%)			
Asian	4 (20.0)	0 (0.0)	4 (10.0)
Black	1 (5.0)	1 (5.0)	2 (5.0)
Indigenous	1 (5.0)	0 (0.0)	1 (2.5)
Other	1 (5.0)	4 (20.0)	5 (12.5)
Unknown	4 (20.0)	8 (40.0)	12 (30.0)
White	9 (45.0)	7 (35.0)	16 (40.0)
Comorbidities—no. (%)			
Cancer	5 (25.0)	4 (20.0)	9 (22.5)
Diabetes	9 (45.0)	11 (55.0)	20 (50.0)
Cardiovascular disease	9 (45.0)	8 (40.0)	17 (42.5)
Chronic kidney disease	6 (30.0)	4 (20.0)	10 (25.0)
Chronic pulmonary disease	3 (15.0)	3 (15.0)	6 (15.0)
Atopy	1 (5.0)	3 (15.0)	4 (10.0)
Charlson comorbidity index			
Median (IQR)	4 (3, 5)	4 (2, 5)	4 (2, 5)
Vaccine status—no. (%)			
Not fully vaccinated (<2 doses)	4 (21.1)	7 (35.0)	11 (28.2)
Fully vaccinated (≥2 doses)	15 (78.9)	13 (65.0)	28 (71.8)
WHO 9-point ordinal scale			
Median (IQR)	4 (4, 5)	4 (4, 5)	4 (4, 5)
Frequencies—no. (%)			
WHO 0/1 (ambulatory: no clinical or virological evidence of infection (0); no limitation of activities (1))	0 (0)	0 (0)	0 (0)
WHO 2 (ambulatory: limitation of activities)	1 (5.0)	0 (0.0)	1 (2.5)
WHO 3 (no oxygen therapy)	3 (15.0)	4 (20.0)	7 (17.5)
WHO 4 (oxygen by mask/nasal prongs)	9 (45.0)	8 (40.0)	17 (42.5)
WHO 5 (noninvasive ventilation/high-flow oxygen)	5 (25.0)	6 (30.0)	11 (27.5)
WHO 6 (intubation/mechanical ventilation)	1 (5.0)	1 (5.0)	2 (5.0)
WHO 7 (ventilation + additional organ support ex./pressors, RRT, ECMO)	1 (5.0)	1 (5.0)	2 (5.0)
Time from symptom onset to randomization—days			
No. of patients	19	19	38
Median (IQR)	10 (5, 16)	9 (4, 14)	10 (4, 15)
Time from admission to randomization—d			
No. of patients	20	20	40
Median (IQR)	2 (1, 4)	1 (1, 2)	2 (1, 2)
Cointerventions—no. (%)			
Dexamethasone	20 (100.0)	19 (95.0)	39 (97.5)
Remdesivir	11 (55.0)	7 (35.0)	18 (45.0)
Tocilizumab	1 (5.3)	0 (0.0)	1 (2.6)
Baricitinib	3 (15.8)	3 (15.0)	6 (15.4)

Abbreviations: ECMO, extracorporeal membrane oxygenation; IQR, interquartile range; RRT, renal replacement therapy; WHO, World Health Organization.

### Primary Outcome

**Figure 2.**
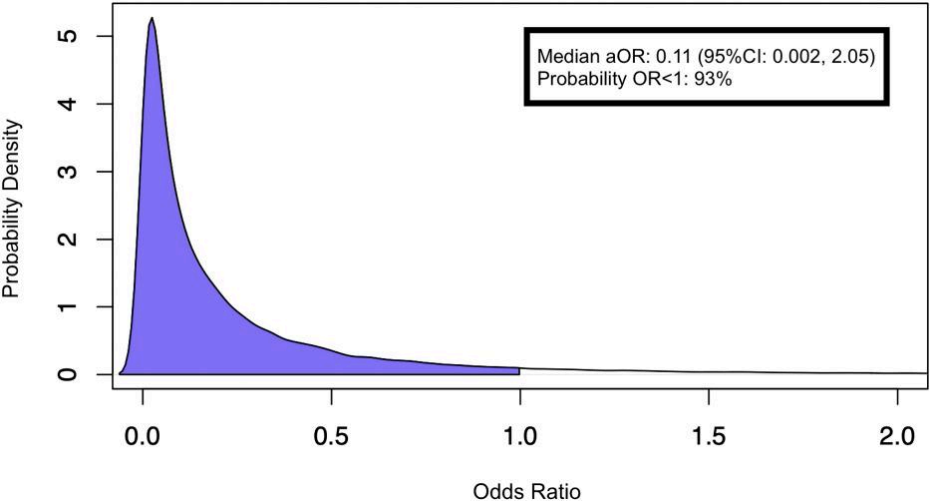
Posterior probability density plot illustrating adjusted odds ratio (aOR) for omalizumab in association with death or mechanical ventilation at day 14.

Follow-up information for the primary outcome was complete for all patients. ​On day 14, 3 (15.0%) patients from the treatment group and 6 (30.0%) from the placebo group had died or received mechanical ventilation. The posterior probability that omalizumab reduced ventilation or death at day 14 was estimated at 92.6% with a median aOR for the primary outcome of 0.11 (95% CrI, 0.002-2.05; Table 2, Figure 2). Sensitivity analysis (without adjusting for covariates) showed similar results (Supplementary Table 2).

**Table 2.** Primary Outcome—Factors Associated With Death or Mechanical Ventilation Through day 14

Parameter	Median Adjusted Odds Ratio (95% Credible Interval)
Age	1.06 (0.96-1.22)
Sex, Female	0.15 (0.003-2.72)
Full vaccination status	0.06 (0.001-1.41)
Intervention, omalizumab, probability of adjusted odds ratio < 1	0.11 (0.002-2.05) 92.6%

### Secondary Outcomes

**Figure 3.**
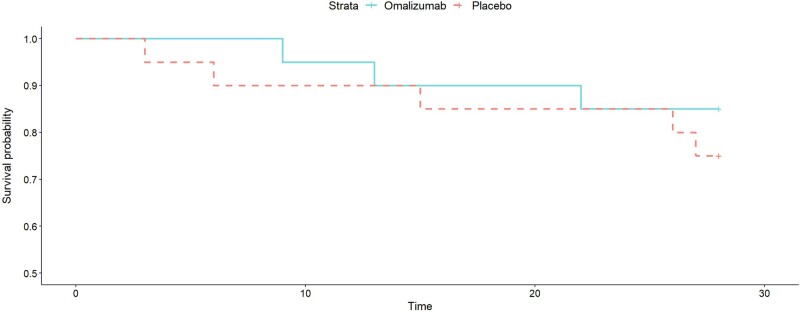
Unadjusted Kaplan-Meier plot depicting all-cause mortality up to 28 d comparing omalizumab and placebo groups.

The probability that omalizumab reduced mortality at day 28 was estimated at 75.9% with an aOR of 0.49 (95% CrI, 0.06-3.90; Table 3). An unadjusted Kaplan-Meier survival curve is shown in Figure 3. Clinical improvement on the WHO scale is reported in Supplementary Table 3 and length of stay in Supplementary Table 4. Omalizumab effect was more notable at day 5 with a 97% probability of clinical improvement with an aOR of 0.02 (95% CrI, 0.00009-1.15). The duration of mechanical ventilation was a preplanned secondary outcome but its calculation was not possible because of the small sample size and therefore is not reported.

**Table 3.** Secondary Outcome—Factors Associated With All-cause Mortality Through day 28

Parameter	Median Adjusted Odds Ratio (95% Credible Interval)
Age	1.05 (0.97-1.15)
Female	0.21 (0.02-1.53)
Full vaccination status	0.79 (0.08-7.95)
Intervention, omalizumab, Probability of adjusted odds ratio < 1	0.49 (0.06-3.90) 75.9%

### Safety and Adverse Events

During the treatment period, a higher number of patients in the control group experienced AEs including SAEs in comparison with patients receiving omalizumab (Table 4). The most common SAE among all patients was acute respiratory distress syndrome, which was numerically more common in the placebo group in comparison to the omalizumab group (25.0% [n = 5] vs 10.0% [n = 2], respectively; Table 4). One patient in each cohort experienced cardiac ischemia, whereas 1 patient experienced cardiac ischemia and arrest in the omalizumab cohort, which was not temporally associated with the administration of omalizumab. None of the SAEs were considered to be related to the study drug in a blinded review. Overall, there were 5 deaths in the omalizumab group and 6 deaths in the placebo group. In the omalizumab group, of the 5 deaths, 1 was due to disease progression and lung infection, 1 death was due to end-stage diffuse large B-cell lymphoma, 1 death was due to end-stage kidney disease and myocardial infarction, and 2 deaths were due to unknown causes (autopsies not conducted) but deemed to be unrelated to the study drug. In the placebo group, 5 deaths were due to COVID-19 progression and 1 death was due to metastatic ovarian cancer. No drug-related AEs such as injection site reactions or anaphylaxis occurred during the study.

**Table 4.** Summary of Adverse Events Reported Through Day 28

Adverse Events	Placebo (n = 20)	Omalizumab (n = 20)	Overall (n = 40)
Mild adverse events			
Injection site reaction	0 (0.0)	0 (0.0)	0 (0.0)
Nausea	2 (10.0)	1 (5.3)	3 (7.7)
Muscle pain	1 (5.0)	0 (0.0)	1 (2.6)
Headache	2 (10.0)	0 (0.0)	2 (5.1)
Joint pain	1 (5.0)	0 (0.0)	1 (2.6)
Severe adverse events			
Anaphylaxis	0 (0.0)	0 (0.0)	0 (0.0)
Acute respiratory distress syndrome			
Moderate	1 (5.0)	1 (5.0)	2 (5.0)
Severe	4 (20.0)	1 (5.0)	5 (12.5)
Cardiac ischemia	1 (5.0)	2 (10.0)	3 (7.5)
Cardiac arrest	0 (0.0)	1 (5.0)	1 (2.5)
Acute kidney injury requiring dialysis	2 (10.0)	1 (5.0)	3 (7.5)
Death	6 (31.6)	5 (27.8)	11 (29.7)

## DISCUSSION

In this multicenter, double-blind phase II RCT of hospitalized patients with laboratory-confirmed SARS-CoV-2 infection and respiratory disease, treatment with omalizumab had a 92.6% probability of reducing the primary outcome of progression to mechanical ventilation or death with an aOR of 0.11 at day 14. Omalizumab also demonstrated a 75.9% probability of reducing all-cause mortality on day 28 with an aOR of 0.49. Numerically, there were fewer SAEs in the omalizumab group and no drug-related SAEs.

Our results support other studies suggesting omalizumab may be beneficial with respect to respiratory viral infection. A study comparing omalizumab with oral antihistamines in children with chronic idiopathic urticaria found that patients treated with omalizumab reported significantly fewer episodes of symptoms consistent with viral upper respiratory tract infections [33]. Similarly, the PROSE RCT compared omalizumab with placebo among children with asthma [34]. Omalizumab significantly reduced viral shedding during respiratory virus–associated exacerbations and diminished both the incidence and duration of viral exacerbations compared with the standard of care alone [35]. In a prospective, observational cohort of ≥150 pediatric asthmatic patients with acute rhinovirus-induced asthma exacerbation, omalizumab (vs supportive care) had a protective effect against mechanical ventilation (OR, 0.17; 95% CrI, 0.04-0.74) and intensive unit admission (OR, 0.31; 95% CrI, 0.12-0.82) [36]. As only a minority of our patients had a preexisting chronic lung disease (3/20 in the omalizumab group, including chronic obstructive pulmonary disease), our study provides the first clinical evidence for a possible antiviral and/or anti-inflammatory effect of omalizumab in nonatopic patients hospitalized for a severe viral respiratory illness.

Nonetheless, these results must be interpreted within the context of the study's limitations, the most notable being that the trial had a smaller than planned sample size because of the premature termination of the study and the 95% CrI for efficacy are wide. Although this trial suggests a reasonable probability that omalizumab may be protective against death and mechanical ventilation in a respiratory viral infection, a larger confirmatory treatment trial would be required.

Nevertheless, this phase II study has several notable strengths, including patient/family and health care worker blinding, complete follow up, and a thorough evaluation for AEs. In light of the need for concrete data regarding the safety of biologic use during a severe respiratory viral infection, the results of this study suggest that omalizumab is safe in patients hospitalized with a severe viral respiratory illness such as COVID-19. The safety of omalizumab is further bolstered by its use with concomitant systemic steroids and Janus kinase inhibitors (eg, baricitinib) in severely ill patients with multiple preexisting comorbidities (including advanced and/or metastatic cancer in 9/40 patients). These results correspond to observational studies that have reported the safe use of omalizumab (for its existing indications) in combination with other biologics and in severe COVID-19 illness [15, 37, 38].

Given its mechanism of action, safety, and practicality (single dose, no known drug interactions, no dose adjustment needed, no laboratory monitoring), omalizumab could be relevant and cost-effective in severe COVID-19 and possibly, other severe respiratory viral infections. Overall, we believe this study could support the development of a phase III program to study the antiviral and/or anti-inflammatory effect of omalizumab in severe respiratory viral illnesses.

## Supplementary Material

ofae102_Supplementary_Data

## Supplementary Data

Supplementary materials are available at *Open Forum Infectious Diseases* online. Consisting of data provided by the authors to benefit the reader, the posted materials are not copyedited and are the sole responsibility of the authors, so questions or comments should be addressed to the corresponding author.

## Notes

***Acknowledgments.*** The authors thank Fonds de Recherche Santé Québec (FRQS), Ministère d’Économie et Innovation (MEI) du Québec, and McGill University Health Centre (MUHC) Foundation for funding this trial. We also thank the patients and their families for their participation in this research. Specials thanks goes to the clinical research team: Hansi Peiris, Karene Proulx-Boucher, Mariam Saleem, Gail Klein and Lisa Patterson.

***Financial support*.** The study was funded by the Ministère de l’Économie et de l'Innovation/Fonds de Recherche Québec Santé and the McGill University Health Centre Foundation. In-kind funding in the form of study drug donation was received from Novartis Pharmaceuticals Corporation.
