# Supplementary material for: COVID-19 Immunologic Antiviral Therapy With Omalizumab (CIAO)—a Randomized Controlled Clinical Trial
Source: Open Forum Infect Dis. 2024 Feb 23;11(4):ofae102. doi: 10.1093/ofid/ofae102 (PMC10977629; doi:10.1093/ofid/ofae102)
Supplement: ofae102_Supplementary_Data [file ofae102_supplementary_data.zip › Supplementary Materials_CIAO_OFID_FINAL.docx]

**Supplementary Materials**

**Supplementary methods**

**Jags model**

model{

# Likelihood

for(i in 1:N){

y[i] ~ dbern(p[i])

logit(p[i]) = beta_0 + beta_age*Age[i] +

beta_sex*Sex_female[i] + beta_Fully_vaccinated*Fully_vaccinated[i] +

beta_treatment*Treatment[i] +

beta_Respiratory_support_1 * Respiratory_support_1[i] +

beta_Respiratory_support_3 * Respiratory_support_3[i] +

beta_Respiratory_support_4 * Respiratory_support_4[i]

# Impute for One Missing Vaccination Status

Fully_vaccinated[i] ~ dbern(p_vaccine)

}

# Prior

p_vaccine ~ dbeta(1,1) # Sensitivity analysis: imputed for a single missing value from beta(11,28). It did not impact the results.

beta_0 ~ dnorm(0,0.0001)

beta_age ~ dnorm(0,0.0001)

beta_sex ~ dnorm(0,0.0001)

beta_Fully_vaccinated ~ dnorm(0,0.0001)

beta_treatment ~ dnorm(0,0.0001)

beta_Respiratory_support_1 ~ dnorm(0,0.0001)

beta_Respiratory_support_3 ~ dnorm(0,0.0001)

beta_Respiratory_support_4 ~ dnorm(0,0.0001)

# Adjusted OR

OR_tr = exp(beta_treatment)

OR_Age = exp(beta_age)

OR_SexFemale = exp(beta_sex)

OR_Fullyvaccinated = exp(beta_Fully_vaccinated)

OR_Respiratory_support_1 = exp(beta_Respiratory_support_1)

OR_Respiratory_support_3 = exp(beta_Respiratory_support_3)

OR_Respiratory_support_4 = exp(beta_Respiratory_support_4)

# Additional

OR_tr_below_one = 1-step(OR_tr-1) # OR<=1

}

**Supplementary Table 1.** Recommended WHO R&D Blueprint expert group Category Ordinal scale for clinical improvement.

**
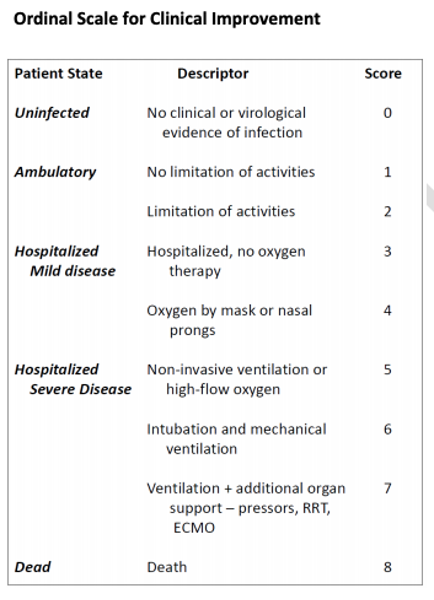
**

**Supplementary Table 2.** Sensitivity analyses (without adjusting for covariates) for primary endpoint (mortality or mechanical ventilation at day 14).

| **Parameter** | **Mean** | **Median** | **Standard Deviation** | **95% CrI** |
| --- | --- | --- | --- | --- |
| OR_tr | 0.544094 | 0.402305 | 0.525575 | 0.07, 1.83 |
| OR_tr_below_one | 0.875417 | 1 | 0.330249 | 1 |

**Legend.** CrI, credible interval; OR, odds ratio; tr, treatment (*i.e.*, omalizumab).

**Supplementary Table 3.** Effect of omalizumab on clinical improvement during various time points from day 1 to day 28.

| **Parameter** | **Median aOR (95% credible interval)** |
| --- | --- |
| **Day 1**  **-Probability of OR < 1** | 0.24 (0.01, 6.04)  80% |
| **Day 5**  **-Probability of OR < 1** | 0.02 (0.00009, 1.15)  97% |
| **Day 14**  **-Probability of OR < 1** | 0.96 (0.05, 19.85)  51% |
| **Day 28**  **-Probability of OR < 1** | 1.10 (0.06, 20.14)  47% |

**Legend.** OR, odds ratio; aORs, adujusted odds ratio.

**Supplementary Table 4.** Factors associated with duration of hospitalization.

| **Parameter** | **Median Adjusted Odds Ratio (95% credible interval)** |
| --- | --- |
| **Age** | 0.0014 (-0.021, 0.024) |
| **Sex, Female** | -0.088 (-0.66, 0.49) |
| **Full vaccination status** | -0.16 (-0.85, 0.53) |
| **Intervention, Omalizumab** | -0.23 (-0.78, 0.32) |
