# Supplementary material for: COVID-19 Immunologic Antiviral Therapy With Omalizumab (CIAO)—a Randomized Controlled Clinical Trial
Source: Open Forum Infect Dis. 2024 Feb 23;11(4):ofae102. doi: 10.1093/ofid/ofae102 (PMC10977629; doi:10.1093/ofid/ofae102)
Supplement: ofae102_Supplementary_Data [file ofae102_supplementary_data.zip › CIAO_Appendix.pdf]

Appendix

COVID-19 Immunologic Antiviral therapy with Omalizumab (CIAO trial) – An Adaptive Phase II Randomized-Controlled Clinical Trial

Short Title: CIAO RCT - Canada

Canadian Sponsor:

Research Institute of the McGill University Health Centre
2155 Guy St, Montreal, Quebec H3H 2L9

Canadian Principal Investigator:

Elena Netchiporouk MD MSc
McGill University Health Centre, Montreal General Hospital, D19-102, 1650 Cedar Ave, Montreal, Quebec H3G 1A4
Assistant Professor of Dermatology

Confidential

STATEMENT OF COMPLIANCE

This protocol will receive independent institutional review board (IRB) permission in each participating site. For the purposes of regulatory compliance, the sponsor institution is the Research Institute of the McGill University Health Center.

SIGNATURE PAGE

The signature below constitutes the approval of this protocol and provides the necessary assurances that this trial will be conducted according to all stipulations of the protocol, including all statements regarding confidentiality, and according to local legal and regulatory requirements and applicable Canadian law and ICH guidelines.

Principal Investigator:

Signed: Elena Netchiporouk ______________________ Date: 05/04/2022 ______________

Elena Netchiporouk
Assistant Professor of Dermatology, Department of Medicine, Research Institute of the McGill University Health Center

PROTOCOL SIGNATURE PAGE

I have read this protocol in its entirety and its appendices. I agree to comply with the requirements of the study protocol and procedures for data recording/reporting and acknowledge my responsibility for the well-being of each research participant, and to ensure that all persons involved in study activities are adequately informed about the protocol, the investigational product, and their trial-related duties. The signature below constitutes the agreement to conduct this study in accordance with the REB approved protocol, GCP and applicable regulatory requirements, including confidentiality, ethical guidelines and regulations regarding the conduct of research in humans.

Qualified Investigator:

Name:
(Print) ____________________________________

Title & Institution:
(Print) ____________________________________

Signature: ____________________________________

Date of signature:
(yyyy-mmm-dd) ____________________________________

COVID-19 Immunologic Antiviral therapy with Omalizumab (CIAO trial)

Contents

1. Key Roles ... 14

2. Background Information and Scientific Rationale ... 15

2.1 Background information ... 15

2.2 Scientific Rationale ... 20

2.3 Potential Risks and Benefits ... 20

2.3.1 Potential risks of Omalizumab ... 20

2.3.2 Potential risk of study procedures ... 22

2.3.3 Potential benefits of Omalizumab ... 23

3. Objectives ... 23

3.1 Study Objectives ... 23

3.2 Study Outcome Measures ... 23

3.2.1 Primary Outcome Measures ... 23

3.2.2 Secondary Outcome Measures ... 23

3.2.3 Exploratory Outcome Measures – Subset of patients in the Montreal area ... 24

4. Study Design ... 25

4.1 Design ... 25

4.2 Study participation duration ... 26

4.3 Study procedures ... 26

4.4 Individually identifiable health information ... 27

5. Study Enrolment and Withdrawal ... 27

5.1 Subject Inclusion Criteria ... 27

5.2 Subject Exclusion Criteria ... 27

5.3 Treatment Assignment Procedures ... 28

5.3.1 Randomization Procedures ... 28

5.3.2 Masking procedures ... 28

5.3.3 Reasons for Withdrawal ... 28

5.3.4 Handling of Withdrawals ... 28

5.3.5 Termination of Study ... 28

6 Study Intervention/Investigational Product ... 29

6.1 Study Product Description ... 29

6.1.1 Acquisition ... 29

6.1.2. Formulation, Packaging and Labelling ... 29

6.1.3 Drug Description 29
6.1.4 Formulation 30
6.1.5 Pharmacokinetics 30
6.1.6 Product Storage and Stability 31

6.2 Dosage, Preparation, and Administration of Investigational Product 31
6.2.1 Drug/Device Handling 31

6.3 Modification of Investigational Product for a Participant 31

6.4 Accountability Procedures for the Investigational Product 31

6.5 Assessment of Subject Compliance 31

6.6 Concomitant Medications/Treatments 32
6.6.1 Permitted medications 32
6.6.2 Prohibited medications 32

7 Study Schedule 32

7.1 Visit and Assessment Schedule 32

7.1 Screening 32

7.2 Enrolment/Baseline 33
7.2.1 Randomization 33

7.3 Main Study Period (Day 0 to Day 14) 33

7.4 Main Study Period Follow Up 33

7.5 Follow-Up Period (6 months) 33

7.6 Early Termination Visit 33

7.7 Unscheduled Visits 34

8 Study Procedures/Evaluations 34

8.1 Visit and Assessment Schedule 34

8.2 Information and Consent Form (ICF) 39

8.3 Clinical and Laboratory Evaluations 39
8.3.1 Demographics 39
8.3.2 Physical Exam 39
8.3.3 Vital Signs 39
8.3.4 Assessment of COVID-19 Disease Severity 39
8.3.5 Laboratory Evaluations 39
8.3.6 Other 40

9 Assessment of Safety 41

9.1 Definitions 41
9.1.1 Adverse Events 42
9.1.2 Serious Adverse Events 42
9.1.3 Unexpected Adverse Events 42

9.1.4 Dose-Limiting Toxicity 42

9.2 Specification of Safety Parameters 42

9.3 Methods and Timing for Assessing, Recording, and Analyzing Safety Parameters 42

9.3.1 Adverse Events 42

9.3.2 Reactogenicity 43

9.2.3 Procedures to be Followed in the Event of Abnormal Laboratory Test Values or Abnormal Clinical Findings 43

9.4 Other Safety Considerations 43

9.4.1 Patient Care: Inpatient and Outpatient and Emergency Department Care 43

9.5 Reporting Procedures 43

9.5.1 Adverse Events 43

9.5.2 Regulatory Reporting 44

9.5.2.1 Reporting to Institutional REB: 44

9.5.2.2 Reporting to Health Canada: 44

9.5.2.3 Reporting to Novartis 45

The following will be reported to Novartis within 15 days of awareness: 45

- All collected SAEs in subjects exposed to omalizumab 45
- All collected pregnancy reports in subjects exposed to omalizumab 45
- All collected reports of abuse and misuse of omalizumab 45

9.5.3 Reporting of Pregnancy 45

9.6 Type and Duration of Follow-up of Subjects after Adverse Events 45

9.7 Safety Oversight (DMSB) 45

10 Clinical Monitoring 45

10.1 Monitoring 45

10.2 Audits and Inspections 46

11 Statistical

Considerations 46

11.1 Study Hypotheses 46

11.2 Sample Size Considerations 46

11.3 Planned Interim Analyses 50

11.4 Final Analysis Plan 50

11.4.1 Primary Endpoints 50

11.4.2 Secondary Endpoints 51

11.4.3 Exploratory Endpoints 51

11.4.4 Handling of Withdrawn Subjects 51

12. Source Documents and Access to Source Data/Documents 51

13. Quality Control and Quality Assurance 52

14. Ethics/Protection of Human Subjects 52

14.1 Ethical Standard 52

14.2 Institutional Review Board........ 52

14.3 Informed Consent Process 52

14.4 Exclusion of Women, Minorities and Children 53

14.5 Subject Confidentiality........ 53

14.6 Futured Use of Stored Specimens 53

15 Data Handling and Record Keeping........ 53

15.1 Data Management Responsibilities........ 53

15.2 Data Capture Methods 53

15.3 Types of Data........ 54

15.4 Timing/Reports........ 54

15.5 Study Records Retention........ 54

15.6 Protocol Deviations........ 54

16 Publication Policy........ 54

17. References 55

List of Abbreviations

AE	Adverse Event
AGA	Allergic Granulomatous Angiitis
ARDS	Acute Respiratory Distress Syndrome
AUC	Area Under the Curve
BSL	Biohazard Safety Level
CBC	Complete Blood Count
CIAO	COVID-19 Immunologic Antiviral therapy with Omalizumab
CDC	Centers for Disease Control
CIU	Chronic Idiopathic Urticaria
COPD	Chronic Obstructive Pulmonary Disease
COVID-19	Coronavirus Disease 2019
CIU	Chronic Idiopathic Urticaria
CT	Computerized Tomography
DSMB	Data Safety Monitoring Board
FcεRI	High Affinity Fcε Receptor
HRV	Human Rhinovirus
ICF	Information and Consent Form
ICU	Intensive Care Unit
ISR	Injection Site Reaction
LTRA	Leukotriene Receptor Antagonist
MERS	Middle East Respiratory Syndrome
REB	Research Ethics Board
RES	Reticuloendothelial System
SAE	Severe Adverse Event
SARS	Severe Acute Respiratory Syndrome
SARS-CoV-2	Severe Acute Respiratory Syndrome Coronavirus 2
SQ	Subcutaneous

Summary

Full Title:	COVID-19 Immunologic Antiviral therapy with Omalizumab (CIAO trial) – An Adaptive Phase II Randomized-Controlled Clinical Trial
Short Title:	CIAO RCT - Canada
Clinical Phase:	II
Sponsor:	Investigator-Initiated Protocol, Research Institute of the McGill University Health Centre
Principal Investigator(s):	Elena Netchiporouk MD MSc FRCPC<br>Co-PI Todd Lee MPH FACP FIDSA
Accrual Ceiling	200
Study Population	Adult patients with confirmed SARS-CoV-2 infection requiring hospitalization (200 patients).
Objective	To evaluate omalizumab's efficacy to reduce severity and all cause mortality of COVID-19 in hospitalized patients.<br>To assess omalizumab's efficacy in reducing post-COVID-19 lung sequelae at 6 months.
Study Design	Phase II, adaptive, 1:1 placebo controlled, double-blind, randomized clinical trial.<br>Primary end point will be assessed at day 14, safety and lung sequelae assessment will be done at 6 months.
Intervention Arm:	Omalizumab 375 mg single subcutaneous (SQ) dose at day 0
Control Arm:	Placebo single SQ dose at day 0
Primary Endpoint(s)	Incidence of mechanical ventilation or death at day 14
Secondary Endpoints	a) time to clinical improvement/hospital discharge (*i.e.,* improvement of 2 points on the 8-category ordinal scale as recommended by the WHO R&D Blueprint expert group (Table 2)<br>b) duration of mechanical ventilation<br>c) confirm safety in COVID-19 patients at day 14

	d) all-cause mortality at day 28
Exploratory Endpoints	a) biomarkers (IgE, D-dimer levels, inflammatory cytokines, pro-fibrotic growth factors)<br>d) viral load<br>e) increased IFN-α response<br>f) presence of lung sequelae at 6 months (*i.e.* spirometry and pulmonary imaging)
Duration of Participation	Primary endpoint will be assessed at Day 14 once 33 patients per study arm will be recruited (interim 1) and 66 patients (interim 2) and final assessment at 100 patients per arm; a study extension will continue for 6 months to assess safety and exploratory endpoints including post-COVID-19 lung sequelae
Inclusion Criteria	1) Positive RT-PCR assay for SARS-CoV-2;<br>2) Disease requiring hospitalization (MUHC respiratory criteria):<br>- Dyspnea at rest or during minimal activity (sitting talking, coughing, swallowing), *OR*<br>- Respiratory rate > 22/min, *OR*<br>- PaO2 < 65mmHg or O2Sat < 90%, *OR*<br>- Infiltrate on chest radiography (CXR) (worsening CXR if baseline abnormal)<br>3) Age ≥18 years;<br>4) Ability to provide consent or consent by proxy.
Exclusion Criteria	Known hypersensitivity to omalizumab or its excipients<br>Receiving Omalizumab or another anti IgE molecule in the last 12 months<br>Inability to give consent<br>Concurrent treatment with a monoclonal antibody targeting following cytokines or their receptors: TNF-alpha, IL-1, IL-4, IL-6, IL-17, IL-23.
Randomization	1:1

Statistical Assumptions	● 2-interim analyses at 33 and 66 patients per treatment arm will be performed<br>● If the posterior probability odds ratio (OR)>0.8 exceeds 0.95 or OR ≤0.6 exceeds 0.8 the trial will be stopped for futility or efficacy, respectively; otherwise it will continue until a sample size of 100 patients per treatment arm is reached (80% power, α=0.05).<br>● The OR will be inferred under the Bayesian framework using a beta-binomial model. The posterior distribution of OR will be obtained by sampling from posterior distribution of risk of death/mechanical ventilation for each arm.<br>● Subgroup analysis for sex/baseline disease severity will be performed.

Schematic of Study Design:

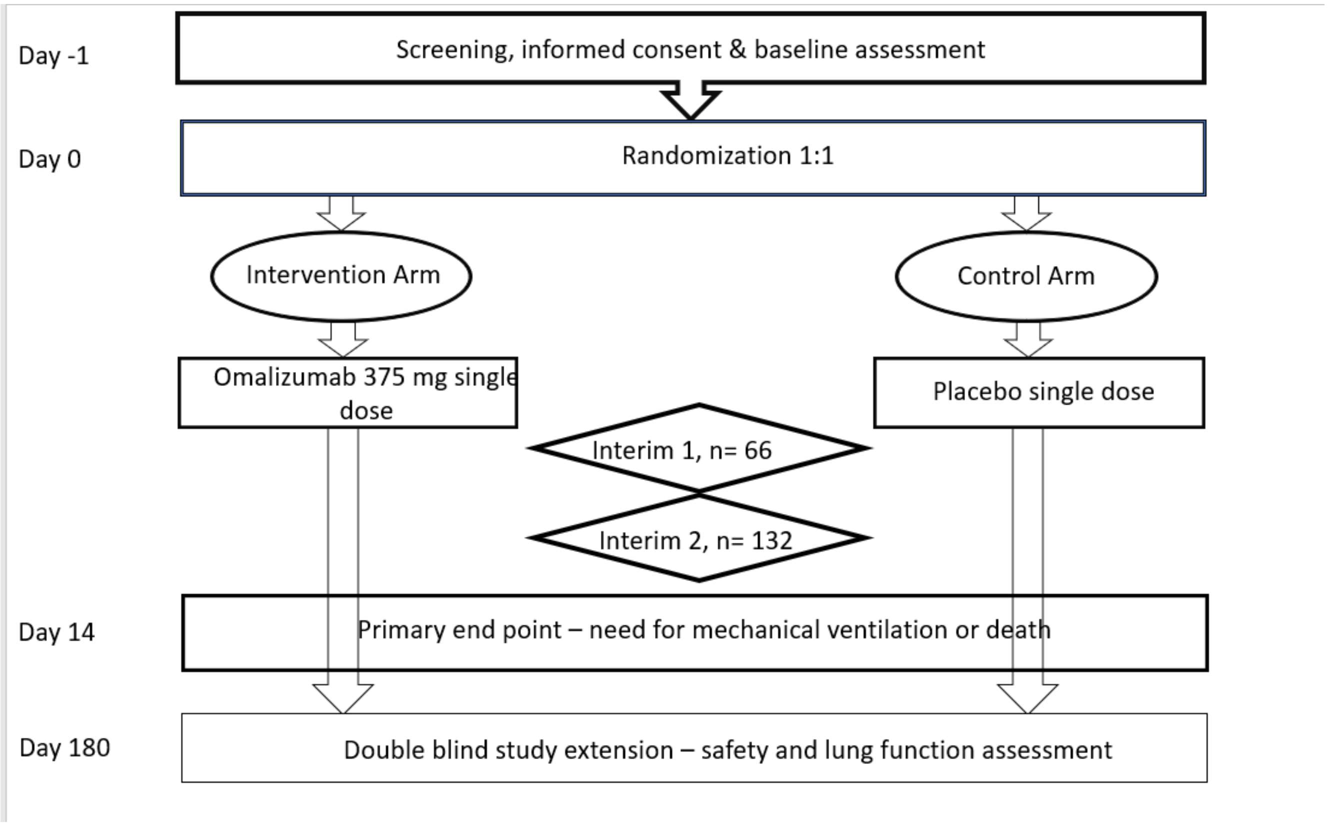

1. Key Roles

Individuals:

Principal Investigator: Elena Netchiporouk MD MSc FRCPC

Co-Principal Investigator: Todd C. Lee MD MPH FIDSA

Co-Investigator: Michelle Le MD

Co-Investigator: Ivan Litvinov MD, PhD

Co-Investigator: Moshe Ben-Shoshan MD, MSc

Co-Investigator: Maxime Cormier MD

Co-Investigator: Ramy Saleh MD

Co-Investigator: Maziar Divangahi, PhD

Co-Investigator: Alexandre Semionov, MD

Co-Investigator: Robert Fowler, MD, MSc

Co-Investigator: Matthew Cheng, MD

Co-investigator: Srinivas Murthy, MD

Study biostatistician: Shirin Golchi PhD

Study biostatistician: Elham Rahme PhD

Research Pharmacist: Kathleen Normandin B. Pharm MSc

Institution: Department of Medicine, McGill University Health Centre (Research Institute), Montreal, QC AND

Centre for Clinical Trial Support (CCTS), Sunnybrook Research Institute

2. Background Information and Scientific Rationale

2.1 Background information

Significance of Research Question and Purpose

Coronavirus disease 2019 (COVID-19) has affected over 137,000,000 people across 216 countries, resulting in the death of almost 3,000,000 individuals and numbers continue to climb exponentially. [1] Despite these staggering numbers, there is currently no effective treatment for COVID-19. As of April 2021, the two most promising therapies are corticosteroids for patients requiring oxygen or ventilation (specifically dexamethasone[2]) and remdesivir for patients requiring oxygen[3, 4].

The immune response to COVID-19 virus appears to follow 2 phases.[5, 6] During the incubation and early disease, interferon (INF-α) signaling and adaptive immunity preclude the disease from progressing.[6] If, and when, this immune response is impaired, the virus may cause massive organ dysfunction leading to Acute Respiratory Distress Syndrome (ARDS).[6] Furthermore, recent evidence highlights the hypercoagulable state of severely ill COVID-19 patients with thromboembolic manifestations potentially affecting any organ.[7, 8] Moreover, several studies have recorded persistent dyspnea, fatigue, psychological and neurological sequelae among recovered COVID-19 patients[9-12].. Imaging studies from Asia demonstrated residual lung abnormalities in COVID-19 infected patients, even in patients who were asymptomatic.[13-15] Similar lung damage has been documented in survivors of Severe Acute Respiratory Syndrome (SARS) and Middle East Respiratory Syndrome (MERS), respiratory diseases caused by coronaviruses similar to SARS-CoV-2, behind COVID-19. Long-term studies of SARS/MERS patients have shown that ~ one third of patients who recovered from a severe infection were left with permanent lung damage 7 months later.[16,17]

Currently, several vaccines are being distributed in Canada to halt the transmission of COVID-19. Among these are two mRNA vaccines produced by Pfizer[18] and Moderna[19], which both boast 94% efficacy in phase III trials, as well as two less efficacious viral vector vaccines. Despite these major successes, mass production and deployment of these vaccines will be a lengthy process. Moreover, the rapid and unpredictable evolution of SARS-CoV-2 may produce vaccine escape variants, rendering currently licenced vaccines ineffective.[20] Indeed, the ChAdOx1 viral vector vaccine is ineffective (10.4% efficacy (95% CI: -76.8%, 54.8%)) against mild to moderate illness caused by the B.1.351 variant of SARS-CoV-2.[21] Therefore, alternative treatments that are non-specific to the strain of SARS-CoV-2 but rather are broadly antiviral and anti-inflammatory are urgently needed to manage severe COVID-19. Several potential rescue drugs under clinical investigation have failed (hydroxychloroquine[2, 3] with/without azithromycin, lopinavir-ritonavir[2, 3], interferon-1-beta[3]) and others like remdesivir have shown benefit for some subgroups but have questionable safety profiles in critically ill patients.[3, 22-24] While the aforementioned drugs aim to treat the virus, the severe pathology seen in ARDS may be related to the host response, instead. Indeed, the best evidence so far comes from dexamethasone[2]. Although other biologics currently under investigation, namely, tocilizumab and sarilumab (IL-6 inhibitors) and baricitinib and ruxolitinib (Janus Kinase (JAK) inhibitors); and IFNs (peg-IFN ƛ-1a and IFN β-1b)[25, 26] have broad spectrum anti-inflammatory properties, they may also dampen viral clearance and increase organ damage.[5] Indeed, completed clinical trials of these biologics have thus far failed to and/or were insufficiently statistically powered to demonstrate efficacy in reducing mortality in COVID-19 patients.[27-31] Moreover, these drugs have a well-documented risk of opportunistic infections, such as tuberculosis, fungal and viral infections.[32,33,34] Furthermore JAK inhibitors have received a black box warning due to their prothrombotic risk, which is

also a problem in COVID-19 patients.[35] While, not associated with infectious risk, IFNs should be used with caution in patients with heart/kidney diseases (common COVID-19 comorbidities) and are associated with bone marrow suppression, hepatitis and neuropsychiatric side-effects[36]. Hence, in addition to the lack of sufficient evidence demonstrating treatment efficacy – the safety profiles of the above mentioned drugs remain to be evaluated in the context of treating COVID-19[37]. Despite the determined efforts, we are still far from eradicating this virus, and preventing mortality and subsequent comorbidities. **New alternative treatments targeting both the virus and the aberrant immune response with a proven safety record in vulnerable and acutely ill patients are urgently needed.**

Existing Literature

Asthma and Chronic Obstructive Pulmonary Disease (COPD) affect 10.8% and 16.6% of Canadians, respectively[38, 39]. It is well known that these patients are at risk of severe disease when infected with respiratory tract viruses such as influenza or rhinovirus (HRV) due to deficient and delayed anti-viral response mediated by INF-α signaling.[40] The largest case series of 44,672 confirmed cases of COVID-19, from China, list **chronic lung disease** among the **top 3** risk factors for **fatal outcome**.[41] In a recently published study from 169 hospitals/8,910 hospitalized patients, the presence of lung disease (*i.e.* **COPD/asthma)** led to a **14.2% mortality rate** *vs.* 5.6% in the absence of lung disease.[42, 43] In a recent meta-analysis, the risk of developing severe COVID-19 infection was 4-fold higher in COPD *vs* non-COPD patients.[41] Furthermore, the Centers for Disease Control (CDC) report of hospitalization notes that patients with chronic lung disease are overrepresented among the younger hospitalized COVID-19 patients. Specifically, **34.6% of all hospitalized patients had chronic lung disease, usually asthma**. When looking by age category: 36.4% of the 18-49 years-old, 28.3% of 50-64 years-old and 38.7% of those ≥65 years and older had chronic lung disease.[44, 45] However, recent data specifically concerning severe asthma patients treated with biologics like omalizumab suggests that these specific patients don't have an increased risk of SARS-CoV-2 or progression to severe forms compared to non-asthmatic population. Among hypothesis involved is the direct implication of omalizumab in stabilizing the effector cells/improving innate immunity to contrast viral infections. [46]

Omalizumab, a humanized anti-IgE antibody, is **approved by Health Canada for the treatment of moderate-severe asthma and chronic idiopathic urticaria (CIU)**.[47] In addition, it has shown promise in **COPD patients <u>with</u> co-existent atopic diathesis** (*e.g.,* atopic dermatitis, hay fever, asthma, eosinophilia).[48] In 2019, >1,3 million people-year received omalizumab (data from Novartis Inc.), where this medication demonstrated an excellent safety record, including in pregnant women and patients with severe comorbidities notably lung disease (*e.g.,* anaphylaxis, asthma (including asthma with coagulopathy), COPD, bronchopulmonary aspergillosis) and cardiovascular disease[49, 50]. Preclinical and clinical data have indicated that omalizumab decreases the hospital stay and the need for mechanical ventilation in the context of virally-induced exacerbations in these patients (Table 1).[51] Further omalizumab efficacy and safety was demonstrated in several non atopic off-label conditions including autoimmune disease (e.g. Behçet's disease, bullous pemphigoid), genetic/immunodeficiency syndromes (e.g. Netherton), severe adverse drug reactions (e.g. toxic epidermal necrolysis).[52] Importantly, the trial of omalizumab in these several conditions was justified by its excellent safety profile and no serious side effects.[52]

Omalizumab binds to the IgE molecule and within 24 hours, it inhibits binding to its high-affinity Fcε receptor (FcεRI) on effector cells and reduces the level of free IgE by 96% within 3 days and FcεRI expression by 73% after 7 days.[47, 53] The importance of IgE-FcεRI interactions in viral infection is supported

by *in vitro* evidence, demonstrating that although IgE-FcεRI interactions heighten susceptibility to viral infections, administration of omalizumab uncouples this interaction and completely abolishes this increased susceptibility[54]. It is well established that patients with asthma are at greater risk of HRV, respiratory syncytial viral and coronaviral infections[55, 56], which is postulated to be due to an impaired IFN-α response. Several studies demonstrated that omalizumab normalizes this impaired antiviral response and subsequently reduces exacerbation frequency[51,57]. Additional clinical data revealed that omalizumab also decreases the duration of HRV and influenza infections, viral shedding, and the risk of HRV-related illnesses in asthmatics via enhancing the IFN-α response[57-60], compared with supportive care alone[58].

In a study of ≥150 pediatric asthmatic patients with acute HRV-induced asthma exacerbation, patients treated with omalizumab *vs* supportive care had 83% (Odds ratio (OR) 0.17, p=0.01) less chance to require mechanical ventilation and 70% (OR 0.31, p =0.01) less chance to need Intensive Care Unit (ICU) admission (Table 1).[51]

Table 1. Omalizumab decreases the hospital stay and need for mechanical ventilation in context of virally induced triggered severe acute asthma exacerbation in patients with asthma. (Ref: Kantor *et al.,* 2016)[51]

Table 2. Omalizumab Is Associated with Reduced Acute Exacerbation Severity

	Univariate Analysis			Multivariate Analysis		
**Outcomes**	**Coef or OR***	**95% CI**	***P* Value**	**Coef or OR**	**95% CI**	***P* Value**
Continuous						
Exacerbation MPIS	−3.32	−4.82 to −1.82	<0.001	−2.83	−4.01 to −1.66	<0.001
Exacerbation PEF%	16.49	8.56 to 24.42	<0.001	13.72	6.99 to 20.45	<0.001
Time to albuterol every 2 h	−15.85	−24.28 to −7.43	<0.001	−16.59	−24.05 to −9.13	<0.001
Hospital length of stay	−24.01	−37.32 to −10.70	0.001	−24.57	−36.17 to −12.98	<0.001
Dichotomous						
Hospital admission	0.38	0.16 to 0.88	0.024	0.30	0.11 to 0.83	0.021
ICU admission	0.31	0.12 to 0.82	0.018	0.24	0.08 to 0.75	0.014
Supplemental O_2	0.26	0.09 to 0.74	0.011	0.24	0.07 to 0.79	0.019
Noninvasive PPV	0.17	0.04 to 0.74	0.035	0.21	0.04 to 1.01	0.052

Definition of abbreviations: CI = confidence interval; Coef = coefficient; ICU = intensive care unit; MPIS = Modified Pulmonary Index Score; OR = odds ratio; PEF = peak expiratory flow; PPV = positive pressure ventilation.
*Coefficient for continous variables and OR for dichotomous variables.

IFN-α was recently demonstrated to have a critical role in suppressing the maladaptive inflammatory response, which causes increased lung damage[61]. In a human bronchial epithelial cell-line model, it has been demonstrated that a single dose of omalizumab is capable of decreasing and preventing inflammation (IL-1β and TNF-α) and secretion of pro-fibrotic growth factors (*e.g.*, TGF- β) responsible for bronchial remodeling and fibrosis within 6 hours.[62]

Figure 1. Reduced IL-1β and TGF-β production with Omalizumab in comparison to control. (Ref : Huang *et al.,* 2005) [62]

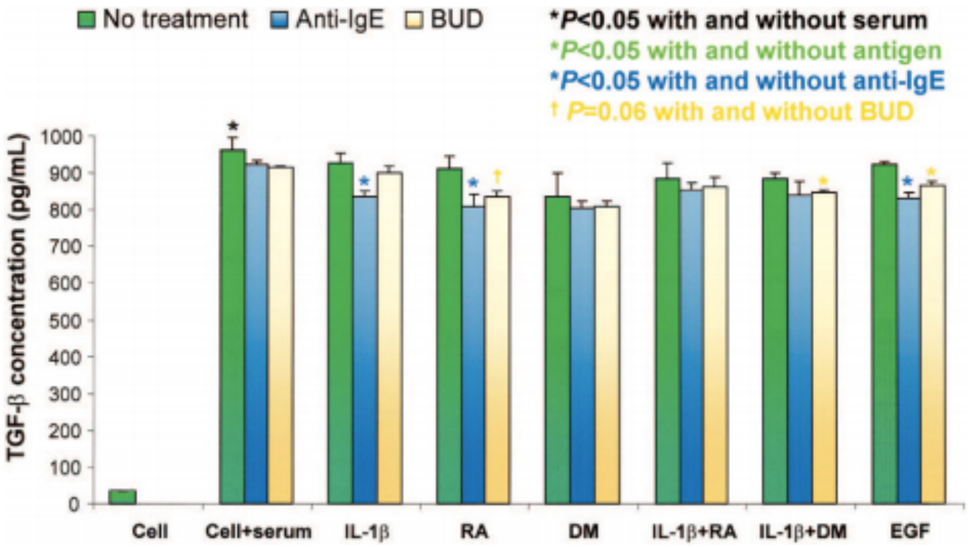

Legend: Mean transforming growth factor (TGF- β) production from bronchial epithelial cells at 6 hours. Atopic serum at 4% for a total IgE concentration of 8 IU/mL was added to all conditions except the cell control. IL-1 indicates interleukin 1; RA, ragweed; DM, dust mite; EGF, epithelial growth factor; and BUD, budesonide. Error bars represent SEM. All samples were tested in quadruplicate. P< 0.05 was considered significant.

In a recent study, omalizumab prevented ARDS and mortality in mice with lipopolysaccharide-induced lung injury in a dose-dependent fashion and decreased markers of inflammation (IL-6, IL-1β and TNF-α) in lung tissue (Figure 2).[63]

Figure 2. Omalizumab improved survival and attenuated lung inflammation in mice suffering from LPS-induced Acute Lung Injury (ALI). (Ref: Wang et al.) [63]

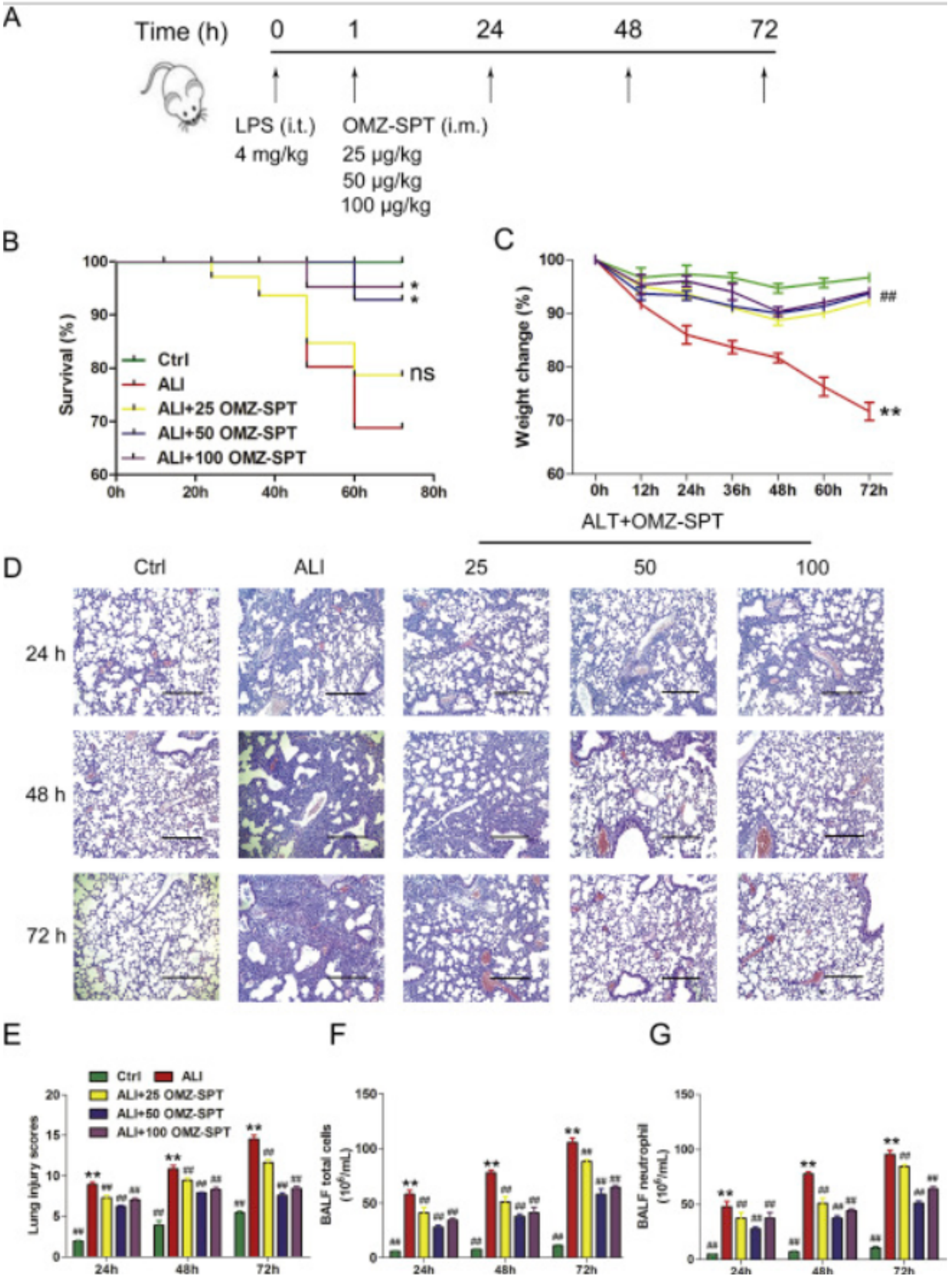

Legend: A. To induce Acute Lung Injury (ALI), mice were stimulated with lipopolysaccharides (LPS). Different doses of omalizumab were administered following LPS. B. Omalizumab prevented death from ALI in a dose-dependent fashion. Kaplan–Meier survival curves. C and D. Similarly, there was a dose

dependent prevention in lung edema. E. Finally, lung injury score (E), total cell count (F) and neutrophil count (G) were reduced in bronchoalveolar lavage fluid (BALF) of mice treated with omalizumab.

Furthermore, knowledge extrapolated from CIU studies highlights the tight interplay between inflammation and the coagulation cascade.[64] Stimulated inflammatory cells and complement activation in CIU trigger the extrinsic coagulation cascade, which in turn worsens vascular permeability and inflammation.[64] Patients with active CIU often demonstrate high levels of prothrombotic factors and/or markers such as D-dimer, prothrombin and factor VII.[65-72] A number of reports, including the data from a recent phase IV omalizumab trial, suggested that D-dimer levels closely correlate with CIU disease activity and improved following omalizumab administration.[73-79] Similar findings were suggested in asthma literature.[77, 78] In COVID-19 patients, acute urticaria and urticarial/morbilliform exanthem are the most common skin findings.[80] A subset of patients that present with skin features suggestive of vaso-occlusive disease have a higher risk of mortality, as reviewed by our research group.[81] Following the recent news stressing the thromboembolic risk related to the COVID-19 infection, a group from China reported that elevated D-dimer levels are capable of predicting COVID-19 related mortality with >90% sensitivity and >80% specificity.[7] Hence, it is possible to postulate that in this setting, Omalizumab may help normalize D-dimer levels in COVID-19 patients, therefore, decreasing the inflammation/coagulation interplay.

Although the aforementioned findings support that omalizumab reduces susceptibility to respiratory viral illnesses and subsequent lung inflammation, whether it exhibits a similar effect on COVID-19 infection is unknown.

2.2 Scientific Rationale

Given that omalizumab has demonstrated **1)** clinical efficacy in reducing severity, hospital stay, and need for mechanical ventilation of virally induced asthma exacerbations, **2)** in vitro evidence for reducing bronchial inflammation, preventing ARDS and reducing lung remodeling and **3)** excellent safety profile including in most vulnerable populations, **we hypothesize that omalizumab could be efficacious and safe in treating adult hospitalized COVID-19 patients**.

Confirming the efficacy of omalizumab in reducing the severity and the burden of COVID-19 infection will help to **1)** improve patients outcomes (survival) and possibly prevent post-COVID-19 lung sequelae; **2)** reduce the burden on the health care system; and **3)** offer a safe and effective treatment for virally induced ARDS beyond the needs of this pandemic.

2.3 Potential Risks and Benefits

2.3.1 Potential risks of Omalizumab

Short and long-term use of omalizumab is well tolerated with an excellent safety record in children, adults, pregnant women, and patients with severe comorbid lung disease. Omalizumab is approved by Health Canada for the treatment of moderate-severe allergic asthma for adults and children above 6 years of age and for the treatment of CIU for adults and children ≥ 12 years-old. Mild adverse drug reactions (AEs) (*i.e.,* malaise, urticaria, dizziness, skin rash) occur in ~8% of patients, and the rate of serious adverse events (SAE) is 1.2%, similar rate to placebo[82-85]. The incidence of the most important SAE, anaphylaxis, is 0.2% and usually occurs in patients with prior history of anaphylaxis.[86, 87]

As per the Product Monograph, the most commonly reported AEs from clinical trials of asthma and CIU (adolescent and adult populations) were mild/moderate and consistent of injection site reactions, viral/respiratory tract infection and headache. In asthma studies, overall AEs were observed with similar rates in Omalizumab treatment groups (85.1%) and control patients (84%). The rate of AEs resulting in clinical intervention, notably discontinuation of Omalizumab or need of medication to treat AEs was observed in ≤0.1 %. Importantly, treatment discontinuation due to AEs was more common in the placebo group (1.4%) vs. Omalizumab-treated patients (0.5%). For example, from asthma clinical trials (947 patients treated with Omalizumab vs. 913 treated with placebo), the AEs occurring in ≥1% of patients regardless of causality assessment were in Omalizumab vs. placebo were: pain (5.4% vs. 4.4%), bone fracture (2.1% vs. 1.1%); leg pain (2.7% vs. 1.5%), dizziness (2.5% vs. 1.5%) and injection site-reactions (45% vs. 43%).

Injections site reactions (ISRs)

Reported presentations included local bruising, redness, warmth, burning, stinging, itching, hive formation, pain, indurations, mass, and inflammation. Most ISRs are mild and do not require treatment alteration. ISRs typically occur within 1 hour of injection and last less than 8 days and do not recur with subsequent injection.

Anaphylaxis

There were only 3 cases (0.08%) of anaphylaxis within 2 hours of Omalizumab administration (vs. 1 control patient (0.05%) in asthma clinical trials. All 3 cases presented with urticaria and throat/tongue edema. Post marketing studies documented the incidence of anaphylaxis of 0.2%.[88]

Idiopathic thrombocytopenia

In a post-marketing study, thrombocytopenia was documented in one patient case.[89]

Allergic granulomatous angiitis (AGA)

There are several case reports documenting AGA with the use of Omalizumab. It is thought that Omalizumab and other anti-asthma medications may unmask AGA in patients with pulmonary disease due to tapering of systemic corticosteroids.[90]

Arthralgia/Myalgia/Joint swelling

Arthralgia, myalgia, and joint swelling is suspected to be an AE relating to Omalizumab, although there have been no definitive cases published in post-marketing studies.

Cardiovascular and Cerebrovascular events

A cohort study of asthmatics comparing 5007 Omalizumab-treated patients versus 2829 patients not treated with Omalizumab indicated higher crude incidence rates of cardiovascular and cerebrovascular SAEs in the Omalizumab-treated group (13.4/1000 person years vs. 8.1/1000 person years in Omalizumab vs. non-Omalizumab, respectively)[91]. Specifically, increased rates were observed for transient ischemic attack (0.7 vs. 0.1 per 1000 person years in Omalizumab vs. non-Omalizumab, respectively), myocardial infarction (2.1 vs. 0.8 per 1000 person years in Omalizumab vs. non-Omalizumab, respectively), pulmonary hypertension (0.5 vs. 0.0 per 1000 person years in Omalizumab vs. non-Omalizumab, respectively), pulmonary embolism/venous thrombosis (3.2 vs. 1.5 per 1000 person

years in Omalizumab vs. non-Omalizumab, respectively) and unstable angina (2.2 vs. 1.4 per 1000 person years in Omalizumab vs. non-Omalizumab, respectively). The rates of incidence of ischemic stroke and cardiovascular death were similar between the two cohorts. The crude rates of arterial thromboembolic events were 6.7 and 4.6 per person years in the Omalizumab and non-Omalizumab treated cohorts, respectively. However, these results may have been confounded by indication as the prevalence of severe asthma in the Omalizumab and non-Omalizumab cohorts was 49.6% and 22.6%, respectively. Moreover, a pooled analysis of 25 randomized, double-blind, placebo-controlled trials comparing the rates of thromboembolic events in Omalizumab-treated patients (n=3342) vs. placebo (n=2895) found similar rates between the two groups[92].

Malignancies

A study comparing 5007 Omalizumab-treated vs. 2829 placebo adolescents/adults followed for 5 years, did not indicate any increase in malignancies.[93, 94]

2.3.2 Potential risk of study procedures

Blood collection

The risks associated with taking blood include possible pain/discomfort from the needle, bleeding, bruising, and infection of the skin. Vasovagal episodes rarely occur. To minimize these risks, an extra blood sample for research will be collected preferentially as part of regular laboratory work up by staff member who is trained to draw blood.

Spirometry

Spirometry can cause coughing or light-headedness, which will go away shortly after the test is finished. The albuterol that is given during reversibility testing can cause increased heart rate and blood pressure, nausea, headache, and a jittery or nervous feeling. These symptoms usually go away in less than an hour.

Medical exams

There are no risks to medical examination, of note no additional physical examinations will be performed by the research team beyond what is clinically indicated as per the standard of care.

Questionnaires

All study related information will be obtained from the treating team/medical chart. Patient will be called by phone to verify demographic information, obtain relevant information to assess for his/her eligibility to take part in the study, explain the study and obtain consent. Participants may refuse to answer any questions that make them feel uncomfortable. Participants' records are carefully protected to respect confidentiality.

Computerized tomography (CT)

The most significant risk of CT imaging of the chest is radiation exposure.[95] Reassuringly, a recent study demonstrated that a single low-dose chest CT scan did not cause statistically significant human chromosomal DNA damage, which would increase cancer risk.[96]

2.3.3 Potential benefits of Omalizumab

- Omalizumab has been judged safe in context of COVID-19 infection by national and international Dermatology and Allergy and Clinical Immunology societies[97, 98]
- There are no reports of severe COVID-19 infection in patients receiving Omalizumab
- Omalizumab is known to improve clinical outcomes in patients who have atopic diathesis including severe asthma and COPD with type 2 inflammation
- It has demonstrated *In vitro* antiviral activity against HRV and influenza viruses, which may present as co-infections with the SARS-CoV-2 virus
- It has demonstrated *In vitro* anti-inflammatory and anti-fibrotic activity responsible for bronchial remodeling and fibrosis as the potential cause of COVID-19 infection lung sequelae[99]
- Omalizumab has showed capable of reducing local nasal mucosal inflammation and improving nasal respiration and improving sinonasal function in patients with chronic rhinosinusitis which may have importance in COVID-19[100]
- Omalizumab has an excellent safety profile and has been safely used in pregnant women and patients with severe comorbidities including lung disease (*e.g.,* asthma, COPD, bronchopulmonary aspergillosis) and cardiovascular disease
- Omalizumab has been demonstrated to be effective for long-term management in patients with severe asthma and other atopic diseases, without increasing the risk of opportunistic infections
- The participants' COVID-19 disease severity may or may not improve during this study. All participants regardless of the study group will receive the standard of care for hospitalized COVID-19 patients in study institutions, as shown Figures 3 and 4 in Section 8.1.

3. Objectives

3.1 Study Objectives

To determine if Omalizumab is effective at treating adult hospitalized COVID-19 patients.

3.2 Study Outcome Measures

3.2.1 Primary Outcome Measures

- Death OR need for mechanical ventilation at day 14.

3.2.2 Secondary Outcome Measures

- Time to clinical improvement (*i.e.,* improvement of 2 points on the 8-category ordinal scale as recommended by the WHO R&D Blueprint expert group (Table 2))
- Duration of mechanical ventilation (among those requiring ventilation)
- Duration of hospitalization

- All cause in hospital mortality at day 28
- Safety in COVID-19 patients at day 14

Table 2. Recommended WHO R&D Blueprint expert group Category Ordinal scale for clinical improvement. (Ref: https://www.who.int/blueprint/priority-diseases/key-action/COVID-19_Treatment_Trial_Design_Master_Protocol_synopsis_Final_18022020.pdf)

Ordinal Scale for Clinical Improvement

Patient State	Descriptor	Score
Uninfected	No clinical or virological evidence of infection	0
Ambulatory	No limitation of activities	1
	Limitation of activities	2
Hospitalized Mild disease	Hospitalized, no oxygen therapy	3
	Oxygen by mask or nasal prongs	4
Hospitalized Severe Disease	Non-invasive ventilation or high-flow oxygen	5
	Intubation and mechanical ventilation	6
	Ventilation + additional organ support – pressors, RRT, ECMO	7
Dead	Death	8

3.2.3 Exploratory Outcome Measures – subset of patients in the Montreal area

- IgE, D-dimer levels
- Inflammatory cytokines levels (*i.e.* TNF-α, IL-1β, IL-6)
- Pro-fibrotic growth factors (*i.e.* TGF-β)
- Viral load (Virologic measures will include the proportions with viral RNA detection over time and viral RNA titer area-under-the-curve (AUC) measurements)
- IFN-α response[101,102]
- Lung sequelae via lung Computed Tomography (CT) and spirometry in consenting patients from both patient arms (omalizumab and placebo).[15,103]

Assessment of clinical outcome measures will be collected by trained research personnel. Exploratory laboratory blood tests will be conducted at McGill University Health Centre Research Institute (Dr. Litvinov laboratory). Computed Tomography scan will be performed in consenting patients and imaging will be read by study radiologist who will be blinded to patients, treatments and outcomes (Dr. Semionov). Pulmonary function test will be done to assess residual lung damage in COVID-19 survivors.

Spirometry will be performed by MUHC-RI spirometry. Results will be interpreted by Dr. Cormier (Respirologist). Both Dr. Cormier and technician will be blinded to patients, treatments and outcomes.

4. Study Design

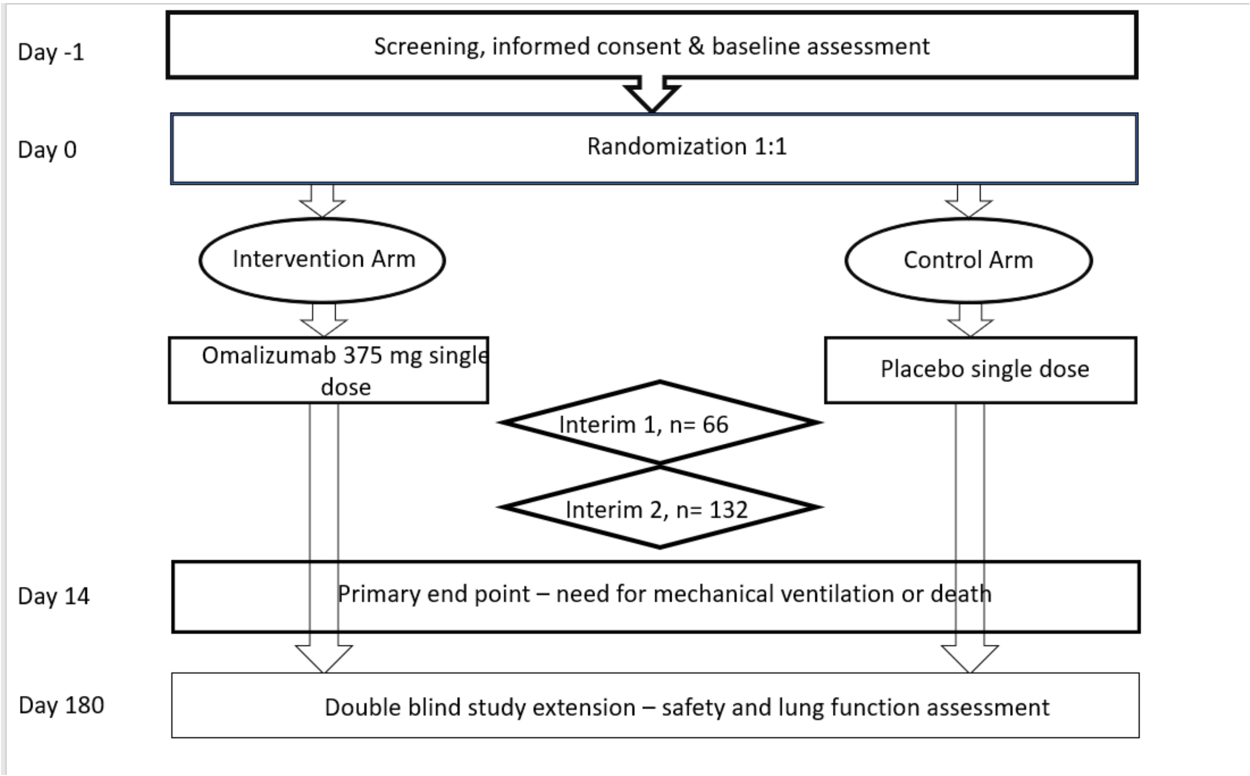

There are two arms: 1) hospitalized COVID-19 patients receiving omalizumab in addition to the standard of care; 2) hospitalized COVID-19 patients receiving placebo in addition to standard of care only. Follow up will occur up to day 14 (+/-3), day 28 (+/-5) for the secondary endpoint and then at 6-months (+/- 14 days) after hospital discharge/disease resolution to assess for lung sequelae.

4.1 Design

Phase II, adaptive, 1:1 placebo controlled, double-blind, randomized.

- Intervention Arm: Omalizumab 375mg subcutaneous injection once. Omalizumab will be provided as 2 prefilled syringes 150mg DIN # 02459795 and 1 prefilled syringe of 75mg DIN #02459787 and may be reconstituted into 1 syringe by research pharmacy at the study site or as per institutional requirements ensuring that blinding is maintained.
- Placebo Arm: Normal Saline matching syringe(s) identical to Omalizumab prepared by research pharmacy.

Because the safety signal in Omalizumab studies is not dose dependent, but preclinical and clinical data suggest that efficacy is dose-dependent, we chose to administer the highest approved dose of omalizumab for asthma patients, *i.e.* 375 mg single subcutaneous (SQ) dose injection.

Omalizumab will be supplied by Novartis pharmaceuticals, free of charge for all study patients. Novartis is NOT the sponsor of this clinical trial and, other than providing the study drug, has no role in the design, conduct, or analysis of this trial which is investigator-initiated.

4.2 Study participation duration

- 28 days (+/- 5) consisting of in-person visits (during hospitalization), laboratory assessments and monitoring for SAE.
- 6-months (+/-14 days) after hospital discharge/disease resolution to assess for lung sequelae (Montreal area patients only).

4.3 Study procedures

4.3.1 Visit and Assessment Schedule

Table 3 provides an overview of the chronological schedule of visits and assessments.

TABLE 3 VISITS AND ASSESSMENT SCHEDULE

**Visit Name**	**Screening~**	**Day 0 (+/-2)**	**Day 1 (+/-2)**	**Day 2 (+/-2)**	**Day 5 (+/-2)**	**Day7 (+/-2)**	**Day 14 (+/-3)**	**Phone call/ visit**	**Day 28 (+/-5) call/visit**	**6-month (+/-14 days) (EOS)/ETV**
								q 1 days for 14 days^		
**Procedure**										
**Informed Consent**	X									
**Inclusion/Exclusion Criteria**	X									
**Demographics**	X									
**Physical Exam***	X	X	X		X		X		x	X
**Vital signs***	X	X	X		X		X		X	X
**Standard investigation as per COVID protocol (Figures 2 and 3)**	X	X	X		X		X			
**Blood sample for research studies****		X		X		X	X			
**Study drug injection**		X								
**Adverse Events Reporting**		X	X		X		X	X	X	X
**PFT and Chest imaging ****										X
**Vital status (alive/deceased)**			X		X		X	X	X	X

~Screening and Day 0 (day of study drug administration) can be done on the same day.
* Physical examination and vital sign intake will be performed as per the standard of care by the treating physician/team and results will be communicated to the study team, no extra examination will be performed for the sole purpose of the study.
** Exploratory analyses will be performed in select study sites (*i.e.* MUHC).
^Phone contact will only occur if the patient has been discharged from the hospital and chart review necessitates clarification from the patient, specifically if patient was discharged prior to day 28, to obtain outcome data. Patient can contact site if they experience any adverse events at any time during the study. The site will contact patient on days 0, 1, 5, 14 and 28 for follow-up to collect outcome data.

4.4 Individually identifiable health information

Patient hospital charts will be consulted, and patients' confidentiality will be respected.

5. Study Enrolment and Withdrawal

The eligibility criteria for this study have been carefully considered. Eligibility criteria are used to ensure that subjects who enter this study are medically appropriate candidates for this therapy. For the safety of the subjects, as well as to ensure that the results of this study can be useful for making treatment decisions regarding other subjects with similar diseases, it is important that no exceptions be made to these criteria for admission to the study.

There will be NO EXCEPTIONS to eligibility requirements at the time of study enrolment. Questions about eligibility criteria should be addressed with the Principle Investigator prior to enrolment.

5.1 Subject Inclusion Criteria

Eligible subjects must meet all of the following inclusion criteria:

1. Positive RT-PCR assay for SARS-CoV-2;
2. COVID-19 disease requiring hospitalization
3. Dyspnea at rest or during minimal activity (sitting, talking, coughing, swallowing), *OR*
   - Respiratory rate > 22/min, *OR*
   - PaO2 < 65mmHg or O2Sat < 90%, *OR*
   - Infiltrate on chest radiography (CXR) (worsening CXR if baseline abnormal)
4. Age ≥18 years;
5. Ability to provide consent or to provide consent via a substitute decision maker

Patients who are pregnant may also be eligible if consent is provided. Patients who have received one of the SARS-CoV2 vaccines and/or bamlanivimab are eligible to participate in the study.

5.2 Subject Exclusion Criteria

Eligible subjects must not meet any of the following exclusion criteria:

1. Known hypersensitivity to Omalizumab or its excipients
2. Inability to give consent themselves or via proxy
3. Patients who received Omalizumab or another anti-IgE molecule in the last 12 months

4. Patients receiving another monoclonal antibody to treat SARS-CoV-2/other indication prior to starting CIAO trial are allowed except if the monoclonal antibody targets following cytokins: TNF-α, IL1, IL4, IL6, IL17, IL23, IL13 or their receptors. However, once the study is initiated, if it is judged by the treating team/patient's doctor that patient's health/clinical status may benefit from a monoclonal antibody (e.g. tocilizumab), this will be allowed.
5. Patients who are below the age of 18

Rationale for inclusion/exclusion criteria

Children rarely need hospitalization for COVID-19 and are even less likely to require mechanical ventilation, there is insufficient clinical data to confirm safety of Omalizumab in children younger than 6 years of age. Hence, children will not be included in our study. Omalizumab is not known to have any meaningful drug-drug interactions and does not need dose adjustment based on age/sex/gender/ethnicity/kidney or liver function. Moreover, omalizumab has found to be safely used in patients who are pregnant. The EXPECT omalizumab post-marketing study (2006-2020) showed no increase in the rate of pregnancy complications or major congenital abnormalities.[104]

5.3 Treatment Assignment Procedures

5.3.1 Randomization Procedures

Eligible patients will be randomly assigned (1:1) to either the omalizumab or the placebo group. Randomization will be stratified according to the level of respiratory support (no oxygen support, oxygen support with nasal duct or mask, non-invasive ventilation, invasive ventilation or extracorporeal membrane oxygenation). The permuted block (4 patients per block-leading to a total number of 100 patients per treatment arm) randomization sequence, including stratification, will be prepared by a biostatistician not involved in the trial using SAS software, version 9.4. Patients will receive medication in individually numbered packs as per randomization order. Unblinding will be permitted only in case of emergency. Study investigators and subjects will be blinded.

5.3.2 Masking procedures

Research pharmacy will supply prefilled syringes identical to omalizumab syringes with normal saline for the placebo control.

5.3.3 Reasons for Withdrawal

Participants may withdraw at any time point at their own discretion.

5.3.4 Handling of Withdrawals

Subjects, who withdraw from the study after signing the ICF, but before receiving the study drug or the placebo, will be replaced. The replacement subject will be sequentially assigned to the current treatment arm with a new subject identification number. If withdrawal occurs once the medication has been opened, no replacement will happen.

5.3.5 Termination of Study

The Qualified Investigator reserves the right to discontinue the study at any time for clinical or administrative reasons. If this study is discontinued for any reason by the Qualified Investigator, REB of study sites and Health Canada will be notified in writing of the discontinuance and the reason(s) why.

If the study is prematurely terminated or suspended for any reason, the Qualified Investigator should promptly inform the enrolled subjects and ensure their appropriate treatment and follow-up.

The end of study is defined as the date on which the last subject completes the last visit (includes the follow up visit).

6 Study Intervention/Investigational Product

6.1 Study Product Description

6.1.1 Acquisition

Omalizumab will be supplied by Novartis pharmaceuticals, free of charge, in prefilled syringes of 75 mg and 150 mg and stored at the research pharmacy as per manufacturer's instructions. All study medication will be kept in a secure place with restricted access and stored in an alarmed refrigerator that is monitored daily. The refrigerator temperature will be at 2-8°C. Central research pharmacy will ship investigational drug to study sites respecting manufacturer instructions.

6.1.2. Formulation, Packaging and Labelling

Omalizumab is supplied as:

Pre-filled syringe (75 mg and 150 mg): Omalizumab is a solution in a single use pre-filled glass syringe with staked needle and rigid needle shield with a Clarity/Opalescence ≤ 30 NTU (Ph.Eur.) and Color ≤ BY5. It is administered as a subcutaneous (SC) injection.

The packaging includes:

- a syringe barrel, 1 mL long, colorless, hydrolytic class I, with staked 26G 1/2" needle. The interior of the barrel and the outer surface of the needle are siliconized.
- a grey plunger stopper, made of latex-free bromobutyl rubber, coated on the product contact side with a fluro resin. The stopper is siliconized.
- a rigid needle shield consisting of a grey styrene butadiene rubber needle shield and a polypropylene rigid shell.

A Omalizumab 75 mg/0.5 mL pre-filled syringe contains 75 mg of the active substance, omalizumab, and the following non-medicinal ingredients: 21.05 mg L-arginine hydrochloride, 1.17 mg L-histidine hydrochloride, 0.68 mg L-histidine and 0.20 mg polysorbate 20 in 0.5 mL water for injection. It is designed to deliver 75 mg omalizumab, in 0.5 mL.

A Omalizumab 150 mg/1 mL pre-filled syringe contains 150 mg of the active substance, omalizumab, and the following non-medicinal ingredients: 42.10 mg L-arginine hydrochloride, 2.34 mg L-histidine hydrochloride, 1.37 mg L-histidine and 0.40 mg polysorbate 20 in 1 mL water for injection. It is designed to deliver 150 mg Omalizumab, in 1 mL.

6.1.3 Drug Description

Omalizumab, a humanized monoclonal anti-IgE antibody.

6.1.4 Formulation

Three prefilled syringes containing 75 mg and 150 mg of Omalizumab, for a total dose of 375 mg may be combined into 1 syringe or as per institutional requirements (ensuring that blinding is maintained) matching the placebo by research pharmacy.

6.1.5 Pharmacokinetics

After SC administration, omalizumab is absorbed with an average absolute bioavailability of 62%. The pharmacokinetics of omalizumab are linear at doses greater than 0.5 mg/kg.

In vitro, Omalizumab forms complexes of limited size with IgE. Precipitating complexes and complexes larger than 1 million daltons in molecular weight are not observed *in vitro* or *in vivo*. Tissue distribution studies in cynomolgus monkeys showed no specific uptake of 125I-Omalizumab by any organ or tissue.

Clearance of Omalizumab involves IgG clearance processes as well as clearance via specific binding and complex formation with its target ligand, IgE. Liver elimination of IgG includes degradation in the liver reticuloendothelial system (RES) and endothelial cells. Intact IgG is also excreted in bile. In studies with mice and monkeys, omalizumab:IgE complexes were eliminated by interactions with FcERI receptor within the RES at rates that were generally faster than IgG clearance.

Asthma

Following a single SC dose in adult and adolescent patients with asthma, omalizumab was absorbed slowly, reaching peak serum concentrations after an average of 7-8 days. Following multiple doses of omalizumab, areas under the serum concentration-time curve from Day 0 to Day 14 at steady state were up to 6-fold of those observed after the first dose.

The apparent volume of distribution of omalizumab in patients with asthma following SC administration was 78 ± 32 mL/kg. In asthma patients omalizumab serum elimination half-life averaged 26 days, with apparent clearance averaging 2.4 ± 1.1 mL/kg/day. Doubling body weight approximately doubled apparent clearance.

The population pharmacokinetics of Omalizumab in asthma patients were analyzed to evaluate the effects of demographic characteristics. Analyses of these limited data suggest that no dose adjustments are necessary for age (6-76 years), race, ethnicity or gender.

Chronic Idiopathic Urticaria

Following a single subcutaneous dose in adult and adolescent patients with CIU, Omalizumab was absorbed slowly, reaching peak serum concentrations after an average of 6 to 8 days. In patients with CIU, Omalizumab exhibited linear pharmacokinetics across the dose range of 75 mg to 600 mg given as a single subcutaneous dose. Following doses of 75 mg, 150 mg or 300 mg every 4 weeks, trough serum concentrations of omalizumab increased proportionally with the dose level. Based on population pharmacokinetic, distribution of omalizumab in CIU patients was similar to that in patients with asthma.

In patients with CIU, based on population pharmacokinetic simulations, omalizumab serum elimination half-life at steady state averaged 24 days and apparent clearance at steady state averaged 260 mL/day (corresponding to 3.3 mL/kg/day for an 80 kg patient).

The effects of demographic covariates and other factors on omalizumab exposure were evaluated using population pharmacokinetics. In addition, covariate effects were evaluated by analyzing the relationship between omalizumab concentrations and clinical responses. These analyses suggest that no dose adjustments are necessary in patients with CIU for age (12 to 75 years), race/ethnicity, gender, body weight, body mass index, baseline IgE, anti-FcεRI autoantibodies or concomitant use of H2 antihistamines or leukotriene receptor antagonists (LTRAs).

6.1.6 Product Storage and Stability

Study drug will be stored in prefilled syringes of 75 mg and 150 mg and stored at the research pharmacy as per manufacturer’s instructions. All study medication will be kept in a secure place with restricted access and stored in an alarmed refrigerator that is monitored daily. The refrigerator temperature will be at 2-8°C.

6.2 Dosage, Preparation, and Administration of Investigational Product

6.2.1 Drug/Device Handling

Omalizumab is classified as a Biohazard Safety Level-1 (BSL-1) supplied in prefilled syringes that are ready for use and does not require special handling or preparation. Prior to injection, pre-filled syringe(s) box(es) needs to be taken out of the refrigerator, date of expiration and consistency (expected to be clear to slightly opalescent) verified and allowed to warm up for about 20 min at room temperature. For blinding purposes, prefilled syringes may be reconstituted into 1 syringe or as per institutional requirements (ensuring that blinding is maintained) matching the placebo prior to administration and sealed in an envelope.

For safety reasons, individuals who meet any of the following criteria should not handle or prepare omalizumab:

1. Individuals with history of allergy to omalizumab.

6.3 Modification of Investigational Product for a Participant

As there is only a single dose of Omalizumab that is administered, there will be no modification to the treatment arm.

6.4 Accountability Procedures for the Investigational Product

The pharmacist at the study site will inventory and acknowledge receipt of all Investigational Product shipments. The IP (and its accompanying documentation) must be kept in a locked area with access restricted to designated staff and stored in accordance with manufacturer’s instructions.

In addition, accurate records will be kept regarding when and how much study product is dispensed and used by each subject in the study. Reasons for departure from the expected dispensing regimen must also be recorded. At the completion of the study, all study products will be reconciled and retained or destroyed as per standard practice at the research pharmacy at each study site.

6.5 Assessment of Subject Compliance

As participants will be given a single SC dose of omalizumab or placebo, participant adherence will not need to be monitored.

6.6 Concomitant Medications/Treatments

Administration of concomitant medications and concomitant therapies by the subject during the study period should be reported and recorded in the patient's case report form. A medication or a therapy is defined as concomitant when administered between the date of signature of the information and consent form and the end of the study.

6.6.1 Permitted medications

Any therapy required to manage symptoms of disease or toxicities related to study therapy. All standard of care procedures for hospitalized SARS-CoV-2 patients will be permitted (*e.g.* dexamethasone).

6.6.2 Prohibited medications

None.

The following should be avoided while on protocol therapy, except where required based on patient's health status.

- Monoclonal antibodies
- Interferon

7 Study Schedule

7.1 Visit and Assessment Schedule

Table 3 in section 4.3.1 provides an overview of the chronological schedule of visits and assessments.

7.1 Screening

Potential subjects will be identified by their treating doctor (covering the COVID-19 ward) and referred to the trial if interested. The trial will be explained in detail by the principal investigator or study personnel and subjects will be given the opportunity to review the consent form. Should patients be interested in participating in the trial, the study coordinator / study personnel will obtain the patient's informed consent. The Qualified Investigator or study personnel will then determine if the subject is eligible for the study. This will be accomplished by reviewing the inclusion and exclusion criteria and completing the screening assessments.

Subjects who do not meet eligibility requirements within the screening period are considered screen failures. Subjects who are screen failures will not be rescreened during the study.

Eligible subjects enrolled in the study will be assigned a subject number that must be used consistently on all documentation and correspondence.

7.2 Enrolment/Baseline

7.2.1 Randomization

Eligible patients will be randomly assigned (1:1) to either the omalizumab or the placebo group. Randomization will be stratified according to the level of respiratory support (no oxygen support, oxygen support with nasal duct or mask, non-invasive ventilation, invasive ventilation or extracorporeal membrane oxygenation). The permuted block (4 patients per block-leading to a total number of 200 patients) randomization sequence, including stratification, will be prepared by a biostatistician not involved in the trial using SAS software, version 9.4. Patients will receive medication in individually numbered packs as per randomization order. Endpoint will be permitted in case of emergency.

Patient screening will be reviewed by central physician. If there are any doubts about the subject's appropriateness, the patient and/or treating team will be called by telephone to obtain more details. All COVID-19 patients hospitalized are treated by expert physicians in Internal Medicine or Respirology. Patients who have an unacceptable medical review will be excluded prior to randomization and the reason noted. Investigational pharmacy will dispense the masked study medicine and the placebo. Study medicine will be administered by trained nursing personnel.

7.3 Main Study Period (Day 0 (+/-2) to Day 14 (+/-3))

The assessments and procedures to be performed during each visit of the main study period are listed in the schedule of Events (Section 7.1). Subjects will receive either a single SQ dose injection of omalizumab or placebo on Day 0. Prior to administration, physical examination and vital sign measurement will be performed. Please refer to Section 7.1 for more details regarding the schedule of events.

7.4 Main Study Period Follow-Up

Each patient will be treated as per WHO COVID-19 protocol by their treating team (please refer to Figure 3A, 3B and 4 for MUHC COVID-19 protocol followed by MUHC and all CATCO sites) who will advise the investigator/study team if any adverse events occurred and on days 0 (+/-2), 1 (+/-2), 5 (+/-2), 14 (+/-3), and 28 (+/-5) and blood samples will be collected for clinical trial monitoring and research in collaboration with MUHC COVID-19 Biobank on days 0 (+/-2), 2 (+/-2), 7(+/-2), and 14 (+/-3). Because of the COVID-19 precautions and limited quantity of protective equipment, most of the follow-ups will be done by phone with the treating team and/or the patient. Please refer to Section 7.1 for more details regarding the schedule of events.

7.5 Follow-Up Period (6 months +/- 14 days)

All patients (still alive) will be followed to day 28 (+/-5) for all-cause mortality. If the hospitalization is longer than 28 days, monitoring of side effects will continue until hospital discharge. In Montreal, subjects will return to the MUHC 6 months after hospital discharge for the last study visit to assess for the presence of COVID-19 sequalae on their lungs (computed tomography and pulmonary function tests).

7.6 Early Termination Visit

Please note that since this is a single dose study there will be no premature discontinuation of treatment.

7.7 Unscheduled Visits

Participants and their treating physicians will be provided contact information of the research team should they have any concerns throughout the study period. This point of contact will be regularly responded 7 days a week in a timely fashion.

8 Study Procedures/Evaluations

8.1 Visit and Assessment Schedule

Table 3 in section 4.3.1 provides an overview of the chronological schedule of visits and assessments.

Figure 3 A and B illustrates the MUHC COVID-19 admission protocol. Figure 3 A and B demonstrates MUHC admission criteria for COVID-19 and current treatment guidelines (this is default care, independent of any study). However, this is an example of standard of care for one of the participating sites. Each hospital will use their own standard of care for COVID-19 patients.

Figure 3. A) MUHC COVID-19 admission protocol

Centre universitaire de santé McGill | McGill University Health Centre

* F M U - 8 6 9 3 *

Prescription d'admission aux soins aigus – COVID-19

COVID-19 - Acute Care Admission Order

Page 1 de/of **2**

ALLERGIES : ________________________________

Poids / Weight ________**kg** **Taille** / Height ________**cm** **Indice de masse corporelle** / BMI ________

**Initiales du prescripteur pour chaque ordonnance** Prescriber's initials for each order	**ORDONNANCE DU MÉDECIN** / PHYSICIAN'S ORDERS	**Initiales de l'infirmier(ère) notées** Nurse's initials noted
Place 'Level of intervention' form in the front of the medical chart		
	**Activity**	
	• Confined to room	
	• Up in chair for meals<br>• Complete CATT form for falls prevention	
	• Plan daily communication with family. Include patient as much as possible.	
	**Vitals Signs and Monitoring:**<br>• Full set of vital signs TID (BP, pulse, RR, O_2 saturation, T°, pain). Notify MD if T° is<br>• CAM q shift and if change in mental status. Notify MD if CAM+ to initiate delirium pre-printed order<br>• Enter weight in OACIS on admission and q week<br>• Capillary blood glucose (CBG) QID if dexamethasone is prescribed	
	O2 via nasal prongs (without humidity) and titrate to keep saturation greater than ☐ 90% or ☐ _________ %. Notify physician if need to increase oxygen.	
	**Nutrition and Hydration (<u>to be ordered in OACIS by MD</u>):**<br>• Diet: ______________________________	
	• Monitor fluid intake. Target intake: ☐ 2L/24h or ☐ __________/24h. Notify MD if pt drinks less than targeted amount.	
	EKG on admission if not done within the last 2 days	
	Insert peripheral IV access with NS lock (see collective order for maintaining patency)	
	**Tests <u>to be ordered in OACIS by MD</u>**<br>• CXR re: COVID-19 on admission if not done within the last 2 days<br>• CBC, INR, PT/PTT,SMA-7 random glucose, urea, creatinine, magnesium, phosphate, total calcium, albumin, LFTs, AST, CRP, d-dimer, troponin, CK, ferritin, LDH **x1 now**<br>• Serum beta-HCG x1 now - *if women of child bearing age who could be pregnant)*<br>• Blood culture, urine culture, sputum culture if T° greater than 38.5°C	
	CBC, SMA-7, troponin ☐ q M-W-F ☐ qTu-Fri ☐ qMonday	

	**Nom en lettres moulées** *Name in print letters*	**Signature**	**N° Permis** *License No.*	**Heure**/*Time* 00:00	**Date** AAYY/MM/JD
**Prescripteur** Prescriber					
	**Nom en lettres moulées et/ou Numéro de permis** *Name in print and letters/or License Number*		**Initiales** / *Initials*	**Heure**/*Time* 00:00	**Date** AAYY/MM/JD
**Infirmier(ère)** *Nurse*					

DM-6212 (REV 2020/10/09) Approbation CPRC (2020/10/02) *CUSM Repro MUHC*

Figure 3. B) MUHC Admission criteria and treatment guidelines

Management of suspected/confirmed COVID-19 cases (adults)

SARS-CoV-2 causes a self-limited influenza-like illness in the majority of cases but can progress to severe lower respiratory tract infection (LRTI), multiorgan failure and death in 2-3% of infected patients. Older age and underlying comorbidities are risk factors for severity.

Excellent supportive care remains the cornerstone of management. This document aims to help clinicians provide appropriate pharmacologic management of suspected cases.

All patients will be offered enrollment in **clinical treatment trials** (standard of care + trial medications) to ensure access to best antiviral and other treatment options. The prescription of investigational or off-label products *outside* of clinical trials (including the use of antivirals, convalescent plasma or host-directed immunotherapy) is **discouraged but may be considered on a case-by-case basis, with ID approval.**

This document will be regularly updated based on new knowledge and information.

Current definition of suspected COVID-19 case: All patients with fever and/or new onset/exacerbation of respiratory symptoms and/or new onset diarrhea

Definition of confirmed COVID-19: Lab detection of SARS-CoV-2 in respiratory sample

Recommended Admission Criteria (use clinical judgment)

- Respiratory criteria:
  - Dyspnea at rest or during minimal activity (sitting, talking, coughing, swallowing), *OR*
  - Respiratory rate > 22/min, *OR*
  - PaO_2 < 65 mm Hg or O_2Sat < 90%, *OR*
  - Infiltrate on CXR (worsening CXR if baseline abnormal)
- Non-respiratory criteria (patient may progress to LRTI):
  - Systolic BP < 100 or signs of sepsis/septic shock, *OR*
  - Altered mental status, *OR*
  - Hematopoietic transplant recipients (HSCT) with high Immunodeficiency Scoring Index ISI*, *OR*
  - HIV with CD4 < 200

***Immunodeficiency Scoring Index (ISI): to be applied *only* for HSCT recipients**

Criterion	ISI
ANC < 500	3
ALC < 200	3
Age > 40	2
Myeloablative conditioning regimen used	1
GVHD, acute or chronic	1
Corticosteroids within the past 30 days	1
Transplant with engraftment < 30 days, or pre-engraftment	1
**Low ISI**	**0-2**
**Moderate ISI**	**3-6**
**High ISI**	**7-12**

Version 5, 2020-09-30

Figure 4. PHARMACOLOGIC MANAGEMENT

No criteria for admission	**Discharge home with instructions for self-quarantine** (as per IPC and Public Health guidelines)<br>**Acetaminophen** 650 mg po q4-6h PRN, max dose of 4 g/day, *avoid if severe hepatic impairment;* offer enrolment in clinical trial *(patient to contact quebec@idtrials.com and MUHC COVID Biobank muhc.covidbb@muhc.mcgill.ca)*
At least one criterion for admission	**Admit** – ***Follow Admission guide for hospitalized patients with COVID-19 (and IPC guidelines for isolation)***<br>***Call coordinator for COVID-19 treatment trials at ext 32146 and ext 23730***<br>• *CATCO (remdesivir vs standard of care to prevent ICU or death; x 32146)*<br>• *CONCOR-1 (convalescent plasma vs standard of care to prevent ICU or death; x 32146)*<br>• *ATTACC (therapeutic vs prophylactic LMWH to prevent ICU or death; x 23730)*
**MILD disease** (O_2 Sat > 92% on no supplemental O_2)	• **Acetaminophen** 650 mg po q4-6h PRN (*avoid if severe hepatic impairment*) AND<br>• **Consider Dalteparin**[2] 5000 units S/C daily if non-ambulatory AND<br>• Offer enrolment in clinical trial
**MODERATE disease** (Need supplemental O_2 to maintain O_2 Sat > 92%)	• **Acetaminophen** 650 mg po q4-6h PRN (*avoid if severe hepatic impairment*) AND<br>• **Dalteparin**[2] 5000 units S/C daily AND<br>• **Dexamethasone**[1] 6 mg po/IV daily x 10 days AND<br>• Offer enrolment in clinical trial<br>• If not candidate for clinical trial AND in **early stage of disease** (< 10 days since onset of symptoms), consider **remdesivir**[3] 200 mg IV x 1 then 100 mg IV (infuse over 120 min) q24h x 4 d (ID approval needed)<br><br>**Antibiotics ONLY if strong suspicion of bacterial superinfection**:**<br>**Ceftriaxone**[4] 2 g IV q24h, reassess in 48 h; max duration 5 days)
**SEVERE disease** (High-flow nasal cannula, invasive or non-invasive mechanical ventilation to maintain O2 Sat > 92%)	****CONSULT ID****<br>• **Acetaminophen** 650 mg po q4-6h PRN (*avoid if severe hepatic impairment*) AND<br>• **Dalteparin**[2] 5000 units S/C daily AND<br>• **Dexamethasone**[1] 6 mg po/IV daily x 10 days AND<br>• Offer enrolment in clinical trial AND<br>• **Piperacillin-tazobactam**[4,5] 4.5 g IV q6h + **Azithromycin**[6] 500 mg po/IV daily - to reassess** in 48 h (max duration 5 days)<br>• Consider Tocilizumab[7] for severe hyper-inflammatory states (case-by-case)<br><br>****Discontinue antibiotics within 48 hours if clinically not deteriorating AND cultures (sputum, blood, sterile specimen) do not reveal a bacterial pathogen**; clinical judgement should prevail over any single lab values (such as WBC, CRP or PCT)<br>Consider adding vancomycin ONLY for patients known to be MRSA colonized/infected; reassess daily

Version 5, 2020-09-30

ADDITIONAL CONSIDERATIONS

[1] **Dexamethasone:**

- Monitor glycemia (CBGM) and adjust glycemic control. If non-diabetic, CBGM for 48-72 hours (risk of new onset hyperglycemia); consider initiating insulin sliding scale if glucose > 10-12
- For patients with severe immunocompromise (HSCT, febrile neutropenia post-chemo, uncontrolled HIV): use of dexamethasone to be determined on a case-by-case basis
- For patients on steroids for another indication: if high dose, can continue same steroid formulation; if low dose, switch to dexamethasone 6 mg po/IV daily
- Dexamethasone can be replaced with Hydrocortisone 50 mg IV q6h

[2] **Dalteparin:**

- Currently insufficient data to support intermediate- or therapeutic-intensity for VTE prophylaxis in seriously ill COVID-19 patients. Follow MUHC Anticoagulation guidelines for dose adjustments according to weight/renal function.

[3] **Remdesivir:**

- Novel antiviral drug, has shown **modest benefit** in hospitalized patients with COVID-19 with reduction in time to recovery. **The supply of this drug is very limited.**
- **Maximal benefits expected in early stages when virus actively replicating** (very low likelihood of significant viral replication after > 10 days of symptoms, and in advanced/severe disease)
- **Effects on mortality, compared to current standard of care, are very uncertain.**
- **Potential adverse events include liver and renal toxicities** – monitor LFTs and Creat daily.

[4] **Hypersensitivity reactions:**

- If type I hypersensitivity to b-lactams: replace pip-tazo/azithro with moxifloxacin 400 mg po/IV q24h
- If type I hypersensitivity to penicillin only, replace pip-tazo with meropenem 1 g IV q8h

[5] **Renal dose adjustment for piperacillin-tazobactam:**

- CrCl 20-40 mL/min: 4.5 g IV every 8 hours; CrCl <20 mL/min: 2.25 g IV every 6 hours; Hemodialysis: 2.25 g IV every 8 hours

[6] **Azithromycin:**

- Risk of QT prolongation and drug interactions - If high-risk of cardiovascular death (several QT prolonging agents, electrolyte imbalance, severe cardiovascular disease), *avoid azithromycin*

[7] **Tocilizumab** (anti-IL6 receptor antibody):

- **Uncertain benefit** in COVID-19. May be considered for extremely severe cases with hyper-inflammatory states (refractory ARDS)
- **Risk of serious bacterial infection and other adverse events** (severe allergic reaction, liver damage/hepatic failure, intestinal perforation have been reported in non-COVID cases).
- Use only on a **case-by-case** basis, in consultation with ID (non-formulary drug form)

Drafted by: M. Semret, M. Cheng, D. Vinh, M. Klein (Division of ID, Department of Medicine), Q. Li and N. Sheehan (Pharmacy Department) - McGill University Health Centre
Reviewed by: S. Qureshi (Resp/ICU), E. McDonald (Internal Medicine), K. Schwartzman (Resp), C. Dupont (Pharmacy), M. Behr (ID)

Version 1 approved March 18, 2020
Version 5: Approved by ASP and P&T Committees on Sep 30, 2020

8.2 Information and Consent Form (ICF)

All subjects must provide consent for the study prior to any study-related procedures including screening assessments. All consent documentation must be in accordance with applicable regulation and Good Clinical Practice. Each subject is requested to sign the ICF after the subject has received and read (or been read) the written subject information and received an explanation of what the study involves, including, but not limited to, the objectives, potential benefits and risks, inconveniences, and the subject's rights and responsibilities. A photocopy of the ICF; all pages and fully executed signature pages must be given to the subject or subject's legally authorized representative, as applicable. Because COVID-19 is communicable illness and study personnel need to be protected against workplace-related disease, local policies may dictate that verbal consent supplants formal written consent and that the patient receives a copy of the paper consent form for their records. Each site will comply with local policies with respect to standards of consent documentation. If the patient's medical condition prevents him/her from providing informed consent, consent from a substitute decision maker will be accepted. If the participant gains the capacity to consent for himself/herself, the consent from a substitute decision maker will end.

The ICF provided to potential subjects must have received favorable opinion/ approval from the REB.

8.3 Clinical and Laboratory Evaluations

8.3.1 Demographics

Demographics, including the subject's race, ethnicity, sex/gender and age, will be documented during the Screening Visit. Underlying medical conditions (*e.g.* diabetes, hypertension), allergies and medications will be recorded.

8.3.2 Physical Exam

Each subject undergoes a full physical exam at hospital admission as per standard of care for COVID-19. All abnormalities will be described in the source documents.

8.3.3 Vital Signs

Vital signs including heart rate, respiratory rate, systolic and diastolic blood pressure, oxygen saturation and temperature are measured every 12 hours or more frequently as per standard of care at each institution.

8.3.4 Assessment of COVID-19 Disease Severity

Performed daily as per institution protocol. Specifically, primary and secondary outcome measures will be assessed such as oxygen saturation, need for mechanical ventilation/Intensive Care Admission, vital status.

8.3.5 Laboratory Evaluations

All laboratory evaluations will be conducted at the institution's Clinical Laboratories as per the standard of care (Figure 3 A and B). Pregnancy status will be ascertained in all eligible female patients of reproductive age and made aware of their pregnancy status prior to signing informed consent form. In addition to the blood collected for clinical needs, in consenting individuals an additional 5cc of blood will be collected for research/exploratory laboratory tests including serum Immunoglobulin E, D-dimer,

cytokine measurement (IL-6, TNF-α, INF-α and TGF-β), growth factors, and viral load at days 0 (+/-2), 2 (+/-2), 7 (+/-2) and 14 (+/-3) in collaboration with the MUHC COVID-19 biobank.

8.3.6 Other

Spirometry and chest imaging (CT scan) will be performed on patients who come for a 6-month (+/-14 days) follow up appointment at the MUHC only.

Table 4 provides a summary of study assessments and Table 5 provides a summary of methodology for exploratory analyses.

TABLE 4 SUMMARY OF STUDY ASSESSMENTS

Investigations		Time in days
Vital Signs,<br>History and Physical Exam	• weight	Screening or at hospital admission
	• blood pressure, heart rate, temperature, oxygen saturation, respiratory rate (assessed by medical personnel)	Every 8 hours or as per protocol
Laboratory investigations as per standard of care	• Electrolytes, urea, creatinine, magnesium, phosphate, total calcium, random glucose, complete blood count (CBC), albumin, liver function tests, C reactive protein, troponin, CK, ferritin, LDH, serum β-HCG in women of childbearing age<br>• CBC, creatinine, electrolytes	Screening<br><br>Every 2-3 days
Additional investigation for study purposes	• Immunoglobulin E (IgE)<br>• D-dimer<br>• Pulmonary function test	0, 2, 7, 14<br><br>180 (6-month)
Translational Research Samples	• Cytokines (.g. TNF-α, IL-1, IL-6)	0, 2, 7, 14
	• Growth factors (.g. TGF-β)	0, 2, 7, 14
	• Viral load	0, 2, 7, 14
	• INF-α levels	0, 2, 7, 14
Radiology	• Computed tomography (CT)	180 (6 months)
Adverse Events	• Daily for 14 days and at 6 months visit	
Vital Status	Day 14 and 28 assessments in hospital or via telephone/video link for patient's vital status. If patient cannot be contacted, consent will have been obtained to: (a) check the electronic medical record; (b) check provincial databases like the dossier santé Québec (c) contact a third-party emergency contact to determine vital status and (d) send a registered letter to their address. All sources will be exhausted	

	before concluding loss to follow up. This technique has achieved 100% follow-up for vital status in Canadian trials of bloodstream infection.

Table 5. Exploratory end-points methodology

Endpoints	Methodology
Viral Clearance<br>(Dr. Divangahi laboratory)	Serial viral load samples will be obtained on day 0 (prior to omalizumab administration) and on days 2, 7 and 14. Serum will be tested for presence of SARS-CoV-2 RNA via real-time quantitative RT-PCR (qRT-PCR) as per protocol previously described by the WHO and Charité Hospital Research Institute https://www.who.int/docs/default-source/coronaviruse/real-time-rt-pcr-assays-for-the-detection-of-sars-cov-2-institut-pasteur-paris.pdf?sfvrsn=3662fcb6_2. Viral RNA detection over time will be measured to determine viral clearance. Additionally, viral RNA titer area-under-the-curve (AUC) will be measured.
Serum IgE, D-dimers, cytokine profiles (TNF-α, IL-6, etc.), IFN-α response, TGF-β response.<br>(Dr. Divangahi and Dr. Litvinov laboratories)	Blood samples will be obtained in all consenting patients on Days 0,2,7,14 and stored in COVID MUHC biobank until all samples are collected. Commercially available ELISA kits will be purchased, and manufacturer's instructions will be followed. Our laboratories are equipped with all the state of the state-of-the-art equipment necessary to conduct these analyses.<br>• Total serum IgE Elisa Kit<br>• D-dimer Elisa Kit<br>• Cytokine 25-plex (includes TNF-α, IL-1-β, INF-α and others) kit<br>• TGF-β Elisa Kit
Reduction of lung pathology 6-months post recovery<br>(Dr. Semionov and Dr. Cormier)	Computed Tomography scan will be performed in consenting patients and imaging will be read by study radiologists who will be blinded to patients, treatments and outcomes (Dr. Semionov). and Pulmonary function test will be done to assess residual lung damage in COVID-19 survivors. Spirometry will be performed using MUHC-RI spirometry. Results will be interpreted by Dr. Cormier.

9 Assessment of Safety

9.1 Definitions

9.1.1 Adverse Events

Any unfavorable or unintended sign, symptom, or disease that is temporally associated with the use of a study agent but is not necessarily caused by the study agent. This includes worsening (e.g., increase in frequency or severity) of preexisting conditions.

9.1.2 Serious Adverse Events

An SAE is defined as an adverse event resulting in any of the following outcomes:

- Death
- Is life-threatening (i.e., an immediate threat to life)
- Prolongation of an existing hospitalization
- Persistent or significant disability/incapacity
- Congenital anomaly/birth defect
- Is medically important*

*Medical and scientific judgment should be exercised in deciding whether expedited reporting is appropriate in other situations, such as important medical events that may not be immediately life-threatening or result in death or result in hospitalization but may jeopardize the patient or may require intervention to prevent one of the other outcomes listed above. These should also usually be considered serious[105].

9.1.3 Unexpected Adverse Events

An unexpected AE is an AE for which the nature or severity is not consistent with the applicable product information (e.g. Investigator's Brochure for an unapproved study agent or package insert/summary of product characteristics for an approved product). Expected means that the event has previously been observed with the study agent and is identified and/or described in the applicable product information. It does not mean that the event is expected with the underlying disease(s) or concomitant medications.

9.1.4 Dose-Limiting Toxicity (DLT)

A DLT is any AE/SAE ≥ Grade 3 as per National Cancer Institute (NCI) Common Terminology Criteria for Adverse Events (CTCAE).

9.2 Specification of Safety Parameters

Omalizumab is a Health Canada approved medicine for the treatment of moderate-severe allergic asthma for adults and children above 6 years of age and for the treatment of CIU for adults and children ≥ 12 years-old, and has proven to be extremely safe throughout several clinical trials in patients with asthma and CIU[106].

9.3 Methods and Timing for Assessing, Recording, and Analyzing Safety Parameters

9.3.1 Adverse Events

Any medical condition that is present at the time that the subject is screened will be considered as baseline and not reported as an AE. However, if the severity of any pre-existing medical condition increases, it should be recorded as an AE. AEs will be captured from randomization for all study arms.

Adverse events (including SAEs) will be recorded and assessed from the time the subject signs the ICF and up to the end of study/ early withdrawal visit. The patient/treating team will be called and asked questions to elicit (any medically related changes in their wellbeing during the hospitalization or up to 28 days. Consenting subjects at the MUHC (which is located in the Montreal Metropolitan area) will be followed for a minimum of 6 months to look for COVID-19 induced lung scarring as side-effects are not expected beyond the 14-day period for omalizumab administration. Thereafter, continued follow-up (in person) is not required except to document ongoing related toxicities until resolved to < grade 2. Adverse events will be classified according to the NCI CTCAE, version 5.0.

Expected adverse events include normal events within the general population, as well as COVID-19 related events which may include pneumonia, ARDS, coagulopathy, sepsis and death[107]. Moreover, expected adverse events related to Omalizumab treatment are listed in section 2.3.1.

9.3.2 Reactogenicity

Not applicable.

9.2.3 Procedures to be Followed in the Event of Abnormal Laboratory Test Values or Abnormal Clinical Findings

AEs identified from any study data (such as laboratory values, physical examinations findings) or from review of health-related documents will be documented in the AE section of the case report form.

9.4 Other Safety Considerations

9.4.1 Patient Care: Inpatient and Outpatient and Emergency Department Care

In the extremely unlikely event of Serious Adverse Event - the lead investigator should be notified immediately. Each participant of this trial will be provided with a wallet card containing the pertinent information regarding the treatments he or she received as part of the trial and contact information (pager) of the study doctor, who can provide additional information. The patient should present this card when he or she is seeking medical care as a hospital inpatient, outpatient and in the emergency department.

9.5 Reporting Procedures

9.5.1 Adverse Events

Adverse events that are assessed and determined **unrelated or unlikely related** to the investigational product will not be recorded and reported. If the site investigator or delegated sub-investigator is unsure about whether the event is caused by or related to the investigational product, then the event will be handled as “related” to the study drug for reporting purposes of the trial. If the causality assessment is

"unknown but not related" to the investigational product, this should be clearly documented in the source documents.

All serious adverse events (regardless of relationship to the investigational product) must be reported in writing and submitted to the CIAO coordinating centre via the CIOMS form/Novartis CT SAE form. Any medication error or inadvertent misuse will be reported irrespective of if a clinical event has occurred.

9.5.2 Regulatory Reporting

9.5.2.1 Reporting to Institutional REB:

Serious adverse events and unanticipated events should be recorded and reported to the REB in accordance with local reporting requirements and timelines.

9.5.2.2 Reporting and Entry Timelines

The investigator is responsible for reporting serious adverse events and serious and unexpected adverse drug reactions (SUADRs) to the CIAO coordinating centre in accordance with applicable regulations and reporting requirements and timelines. The CIAO coordinating centre will then report to the Sponsor.

Study investigators will report SAEs to the CIAO coordinating centre within the following timelines:

- All deaths and immediately life-threatening events will be recorded and reported to the sponsor within 24 hours of site awareness.

- Serious adverse events other than death and immediately life-threatening events, regardless of relationship, will be reported to the sponsor within 72 hours of site awareness.

Adverse event information will be entered into the CRF in a timely manner and **no later than 15 days** from the time the investigator becomes aware of the event.

Serious adverse event information will be entered into the CRF in a timely/**within 72 hours** from the time the investigator becomes aware of the event.

9.5.2.3 Sponsor Reporting to Health Canada:

The regulatory sponsor is responsible for reporting SUADRs to regulatory authorities in accordance with local expedited reporting requirements and timelines. In addition, the Sponsor will complete the ADR Expedited Reporting Summary Form and submit this form to the appropriate Health Canada directorate.

Serious, unexpected, adverse drug reactions will be reported according to the following criteria:

a) Where it is neither fatal nor life threatening, within 15 days after becoming aware of the information.

b) Where it is fatal or life threatening, immediately where possible, and, in any event within 7 days after becoming aware of the information.

Relevant follow up information to safety report will be submitted as soon as the information is available.

Upon request from regulatory authority, the sponsor will submit any additional data or information that the agency deems necessary, as soon as possible, but in no case later than 15 calendar days after receiving the request.

9.5.2.4 Reporting to Novartis

The following will be reported to Novartis within 15 days of awareness:

- All collected SAEs in subjects exposed to omalizumab
- All collected pregnancy reports in subjects exposed to omalizumab
- All collected reports of abuse and misuse of omalizumab

9.5.3 Reporting of Pregnancy

A pregnancy will be documented for follow-up purposes. All pregnancies that are identified during the study after the Screening Visit will be followed to conclusion and the outcome of each will be reported. If the pregnancy results in anything other than a normal birth or elective abortion of a healthy fetus, it will be reported as a serious adverse event to the Health Canada.

9.6 Type and Duration of Follow-up of Subjects after Adverse Events

All subjects will be followed for a minimum of 6 months (+/-14 days) to look for COVID-19 induced lung fibrosis (via spirometry and CT) as side-effects are not expected beyond the 14-day period for Omalizumab administration. Thereafter, continued follow-up (in person) is not required except to document ongoing related toxicities until resolved to < grade 2.

9.7 Safety Oversight (DSMB)

Data Safety Monitoring Board (DSMB) will be established and will include members who are experts in the field to review all the clinical trial data and adverse events. The DSMB would also review all results of any interim analysis.

10 Clinical Monitoring

10.1 Monitoring

Monitoring will occur throughout the trial by an individual who is not involved in data collection. The extent and nature of monitoring is outlined in the Monitoring Plan. The monitoring plan specifies the frequency of monitoring, monitoring procedures, the level of site monitoring activities (e.g., the percentage of participant data to be reviewed), and the distribution of monitoring reports. The monitor will seek evidence that each individual involved in the trial has adequate qualifications, training on the protocol, and that study resources remain adequate throughout the trial period. The monitor will seek evidence that each site’s regulatory files containing GCP essential documents are kept current. Evidence that the study protocol and any amendments, the Investigator’s Brochure and any amendments, and the study informed consent form have all received appropriate REB approvals and that the approvals have been filed with the regulatory documents. The monitor will generate a site monitoring report for the investigator to detail significant findings, deviations, deficiencies, plausibility, record completeness and any corrective actions to be taken by the site investigator and research personnel. Copies of these reports will be provided to the study site upon completion of the monitoring assessment.

The REDCap database system will enable complete record keeping.

10.2 Audits and Inspections

This study may be subject to a Quality Assurance audit during the course of the study. The Quality Assurance (sponsor) may conduct audits of clinical research activities in accordance with internal standard operating procedures to evaluate compliance with the principles of GCP and ICH related guidelines. In addition, inspections may be conducted (during the study or after its completion) by Health Canada, at their discretion.

The Investigator will permit such audits by the Quality Assurance department or Health Authorities and facilitate them by providing access to the relevant source documents.

11 Statistical Considerations

11.1 Study Hypotheses

We hypothesize that a 375mg single dose of Omalizumab is more efficacious than placebo in decreasing hospitalisation duration, need for mechanical ventilation and all-cause mortality in hospitalized patients with COVID-19, in whom the safety and efficacy of Omalizumab has been confirmed.

11.2 Sample Size Considerations

The maximum allowable sample size will consist of 200 patients. However, the final trial size is a random variable as the design allows for early stopping at one of two interim analyses. The total sample size and stopping criteria are specified to achieve 80% power.

Operating characteristics of Bayesian adaptive design for CIAO trial

The placebo controlled adaptive design allows for the following possibilities at (equally spaced) interim look(s):

- Stop the trial for superiority if the posterior probability of the OR being less than a pre-specified value (representing efficacy) crosses a pre-specified threshold, i.e.,

$$P(\mathrm{OR} < \mathrm{OR}_{\mathrm{eff}}) > u_{\mathrm{sup}}$$

- Stop the trial for futility if the posterior probability of the OR being greater than a pre-specified value crosses a pre-specified threshold, i.e.,

$$P(\mathrm{OR} > \mathrm{OR}_{\mathrm{eff}}) > u_{\mathrm{fut}}$$

- Continue the trial to the next interim/maximum sample size, N.

Trial operating characteristics, including power (defined as probability of stopping for superiority), false positive rate (defined as probability of superiority when OR = 1), and probability of concluding futility are explored for the following through the following simulation scenarios:

OR	0.3, 0.4, 0.5, 0.6, 0.8, 1
u (superiority threshold)	0.8, 0.85, 0.9, 0.95
$\mathrm{OR}_{\mathrm{eff}}$	0.6, 0.8
Number of interim looks	1, 2

The futility threshold is held fixed at $u_{\mathrm{fut}} = 0.95$ and the control risk is $p_0 = 0.35$. The sample size is $N = 200$ patients randomized into the two arms. The interim looks are at about 50 patients per arm for the 1-interim design and at about 33 patients per arm for the 2-interim design.

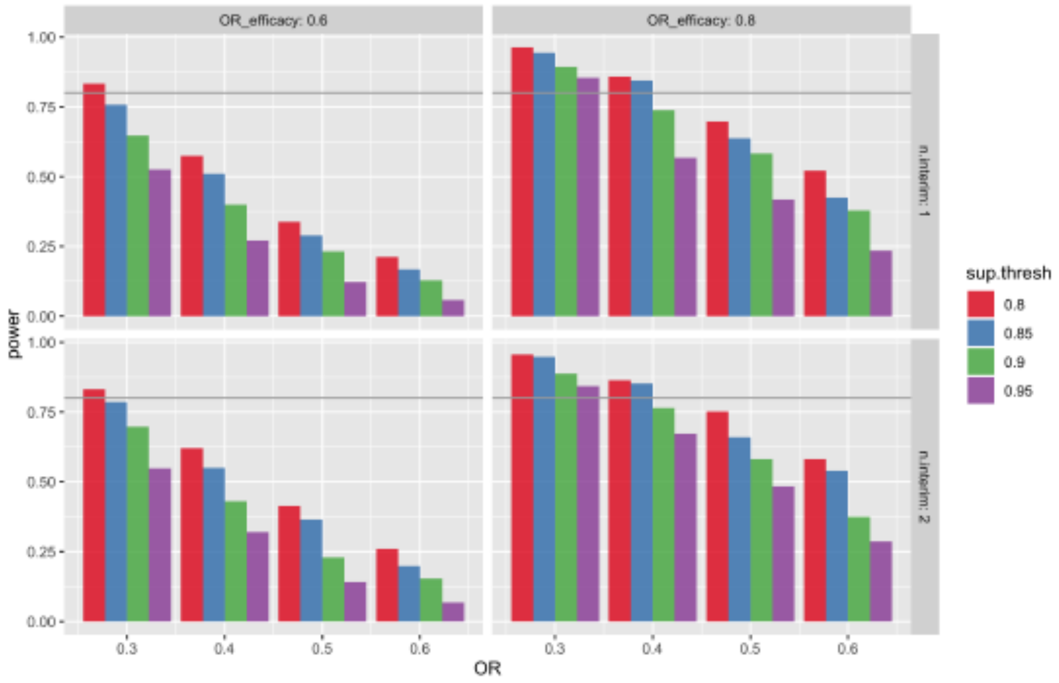

Figure i: The height of the bars shows power, i.e., probability of concluding superiority at any point (interim or final analysis) for any of the simulation scenarios. The grey line shows 80% power.

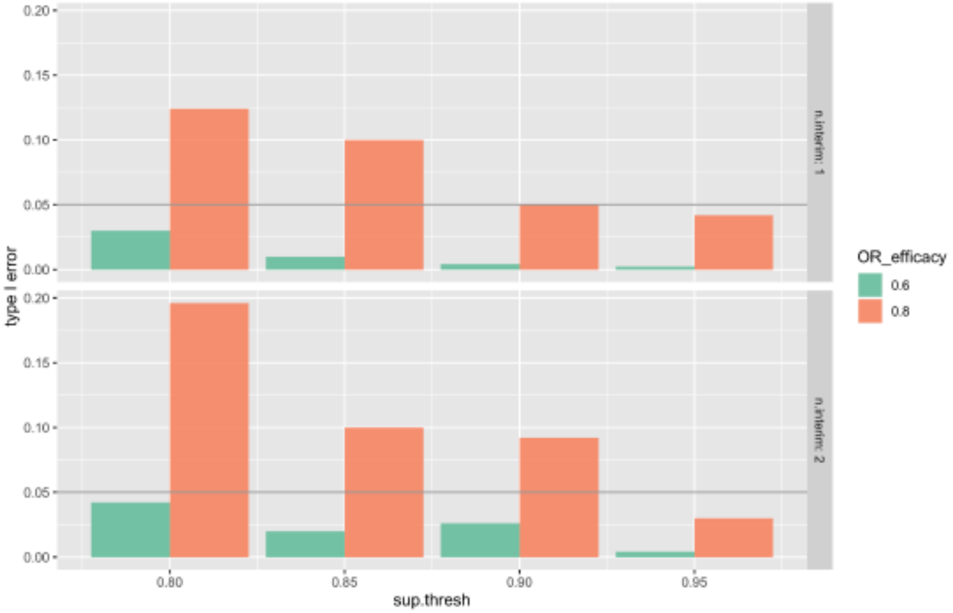

Figure ii: The height of the bars shows false positive rate, i.e., probability of concluding superiority at any point (interim or final analysis) given that OR is actually 1 for any of the simulation scenarios. The grey line shows 5% false positive rate.

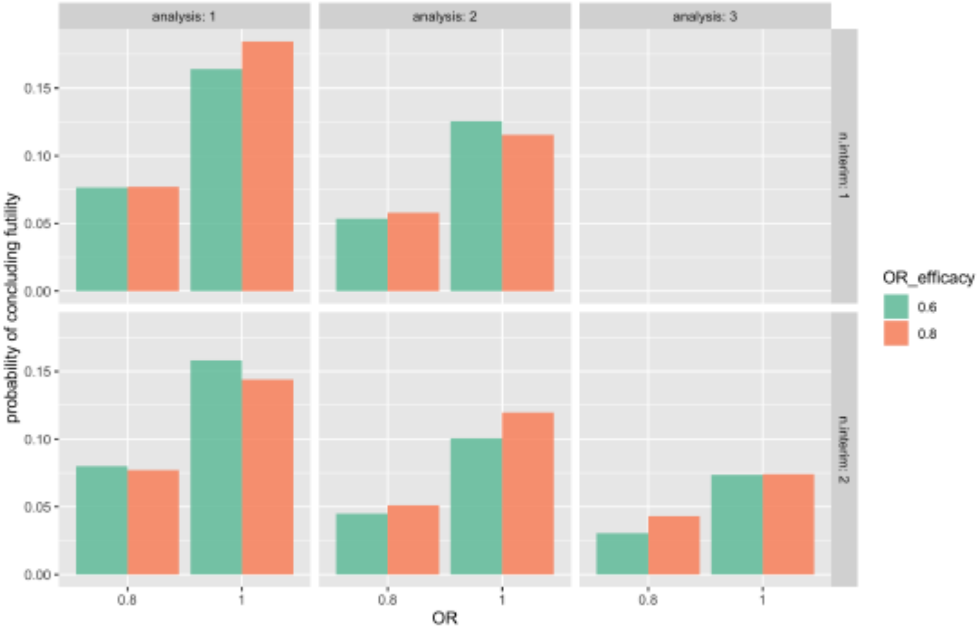

Figure iii: The height of the bars shows probability of concluding futility by interim analysis given that the trial is in fact futile, i.e. OR is either 0.8 or 1 for any of the simulation scenarios.

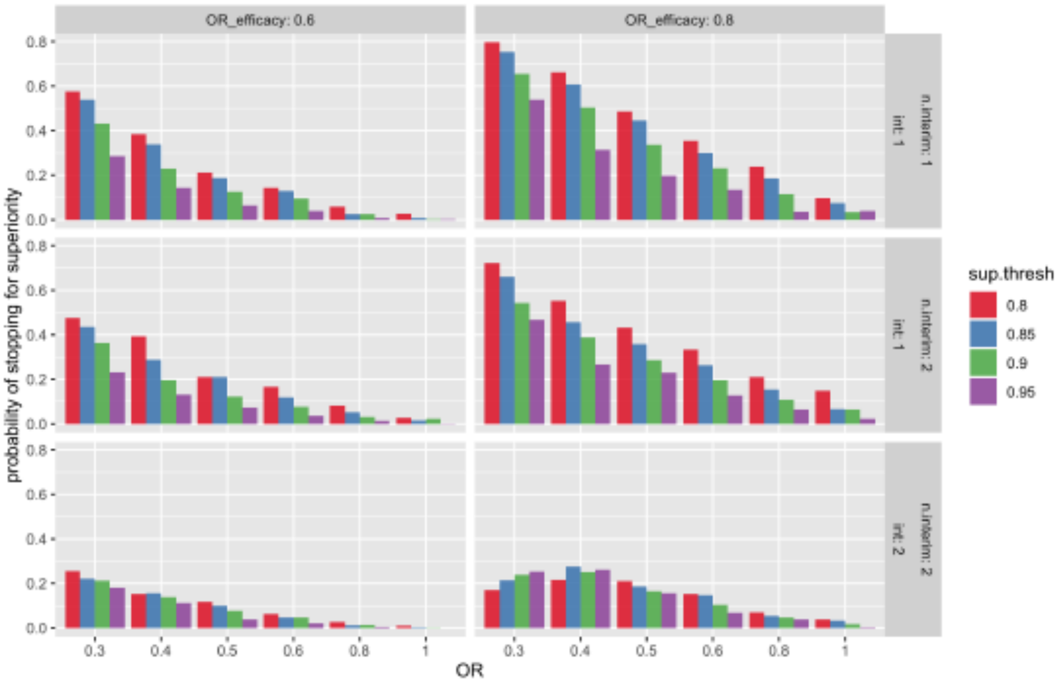

Figure iv: The height of the bars shows probability of stopping early at an interim analysis due to superiority for various scenarios.

11.3 Planned Interim Analyses

Two interim analyses for the primary outcome will be performed (at 33 & 66 patients per arm). If the posterior probability that OR>0.8 exceeds 0.95 or posterior probability that OR ≤0.6 exceeds 0.8 the trial will be stopped for futility or efficacy, respectively; otherwise, it will continue until a sample size of 100 patients per treatment arm is reached (overall roughly equivalent to a frequentist 80% power, α=0.05).

11.4 Final Analysis Plan

11.4.1 Primary Endpoints

The primary endpoint, incidence of mechanical ventilation or death at day 14, will be calculated (number of cases/ person-days) as OR of mechanical ventilation and/or /death with 95% credible intervals. The OR will be inferred under the Bayesian framework using a beta-binomial model. The posterior distribution of OR will be obtained by sampling from posterior distribution of risk of death for each armin an intention-to-treat basis to compare response rates between treatment arms accounting for stratification for baseline respiratory status. Potential confounders (e.g. concomitant COVID-19 treatments) will be taken into consideration in statistical analysis.

11.4.2 Secondary Endpoints

Linear mixed model will be used for secondary end-points such as duration of hospitalization and duration of mechanical ventilation. Time-to-event distributions for the overall in-hospital survival will be estimated in all subjects using the Kaplan-Meier estimate. Comparison of adverse events and descriptive characteristics will also be performed. Potential confounders will be taken into consideration in statistical analysis.

11.4.3 Exploratory Endpoints

Subgroup analyses will be performed for sex/gender, baseline d-dimer and IgE levels and pre-existing comorbidities for recruitment. Differences in continuous variables (e.g. cytokine levels, duration of mechanical ventilation, etc.) will be calculated using Hodges-Lehmann estimation. Additionally, viral RNA titer area-under-the-curve (AUC) will be measured.

11.4.4 Handling of Withdrawn Subjects

If a subject withdraws from the study after receiving omalizumab, the subject will not be replaced. Subjects who withdraw from the study after signing the ICF, but before receiving omalizumab, will be replaced. The replacement subject will be sequentially assigned with a new subject number.

11.5 Co-Enrolment in Separate Clinical Trials Considerations

Co-enrolment in other clinical trials will be accounted for by adding interaction terms to the regression model.

11.6 Timing of Study Drug Administration Considerations

The variability in the timing of administration of the study drug in the disease course of COVID-19 will be taken into account by adding the number of days since initial symptoms to the regression model. Importantly, respiratory status at baseline, sex and comorbidities will be accounted in the regression model as well.

11.7 Pulmonary Sequelae at 6 Months Considerations

Where available, baseline imaging and pulmonary function results will be obtained from patients' medical records to adjust for baseline lung disease differences or any other baseline measure for those who have this data and we will treat the rest as missing data by data imputation or data augmentation.

12. Source Documents and Access to Source Data/Documents

Source documents will include documentation of the patients' clinical status and investigations, such as blood tests and imaging results during the patient's admission that will be obtained by the clinical research nurse.

Participants will be asked to provide consent to obtain medical records from their healthcare provider or public health official, if there is the need to verify any changes in the participants' clinical status or if participants experience an adverse event.

13. Quality Control and Quality Assurance

Study medications will be Health Canada-approved following Good Manufacturing Practice.

14. Ethics/Protection of Human Subjects

14.1 Ethical Standard

The Investigator will ensure that this study is conducted in full conformance with ICH/GCP Guidelines, the principles of the Declaration of Helsinki and with local laws and regulations in which clinical research is conducted. All investigators must have received ethics and GCP training prior to human subject involvement.

14.2 Institutional Review Board

The Investigator will submit this protocol and any related documents provided to the subject (such as subject information used to obtain informed consent) to the REB of MUHC. Approval from the REB must be obtained before starting the study and should be documented in a dated letter to the Investigator, clearly identifying the trial, the documents reviewed and the date of approval.

Modifications made to the protocol after receipt of the REB approval must also be submitted as amendments by the Investigator to the REB of MUHC in accordance with local procedures and regulations.

A Health Canada Research Ethics Board Attestation form must be completed and signed by the Research Ethics Board representative. Alternatively, an attestation that the EC/IRB follows and complies with Part C Division 5 of the Food and Drug. Annual re-approval is required for as long as subjects are being followed on protocol.

Amendments: All protocol amendments will be confirmed in writing and submitted, as appropriate, for review by the Health Canada and the REB. No protocol modifications can be implemented prior to the approval of the amendment by Health Canada and the REB unless in exceptional urgent situation for the safety of the subjects.

14.3 Informed Consent Process

It is the responsibility of the Investigator to obtain written OR given constraints of COVID-19 isolation a non-written informed consent obtained through reading the contents of the informed consent form to the trial participant (or substitute decision maker) and receiving the individual's informed consent before a witness, and subsequent attestation by the witness that the consent was given informed consent from each individual participating in this study after adequate explanation of the aims, methods, objectives and potential hazards of the study. Subject consent must be obtained according to EC/IRB requirements prior to any study-specific procedures. The Investigator must also explain to the subjects that they are completely free to refuse to enter the study or to withdraw from it at any time for any reason without penalty or loss of benefits to which they are otherwise entitled. Appropriate forms for documenting written informed consent will be provided to the sites prior to the study.

The Informed Consent Form will be provided in English or French at the choice of the subject.

14.4 Exclusion of Women, Minorities and Children

- Persons under the age of 18 are not eligible to participate.
- Non-English or French speaking adults are not eligible as the consent forms will be only available in English or French.

14.5 Subject Confidentiality

- Documentation, data and all other information generated will be held in strict confidence. No information concerning the study, or the data will be released to any unauthorized third party, without prior written approval of the participant, except as necessary for monitoring by the IRB or public health authorities.
- No participant identifying information will be disclosed in any publication or at any conference activities arising from the study.
- Clinical data will be entered into a study specific database by designated staff on a regular basis from completed RedCAP electronic Case Record Forms (eCRF). Access to database will be given to authorized personnel only (members of the immediate study team). eCRF and trial documents will be kept in a secure database.

14.6 Future Use of Stored Specimens

Participants will be asked for consent to collect blood for research and to use residual specimens for secondary research. In consenting individuals extra blood will be drawn for correlatory studies on days 0, 2, 7, and 14 (days of MUHC standard COVID-19 biobanking). All samples will be de-identified and labelled to preserve confidentiality. Samples will not be sold for commercial profit. Participants will have no legal or financial interest in any commercial development resulting from any future research. There are no direct benefits to the subject for extra specimens collected or from the secondary research. Subjects may withdraw permission to use samples for secondary use at any time. They will need to contact the study site and the samples will be removed from the study repository after this study is completed and documentation will be completed that outlines the reason for withdrawal of permission for secondary use of samples.

15.1 Data Handling and Record Keeping

15.1 Data Management Responsibilities

Investigators will maintain a secure REDCap database of study records at the study institution.

15.2 Data Capture Methods

Data will be collected by the clinical nurse on the research team while the patient is admitted in the hospital. This information will then be inputted into the REDCap database study records by the study coordinator / study personnel. Data recorded will be password protected and only accessible by the research team. The patient's identity will be protected by replacing their name with a research number. Only the research team will have access to the code linking the patient's name to this number.

15.3 Types of Data

Participants and/or the treating team (with patient consent) will be asked to provide demographic information, respiratory/vital status, access to relevant laboratory investigations and imaging results during the study follow-up period.

15.4 Timing/Reports

- An enrollment progress report will be generated monthly
  - Participants Enrolled
  - Participants on study
  - Participants completed the study
  - Lost to Follow Up
  - Reported outcomes (clinical and investigational - pooled, both arms)
- De-identified data will be shared with the research team members for analysis.

15.5 Study Records Retention

- Paper documents will be stored in a locked cabinet, in a secure and locked office (Dr. Netchiporouk) for up to 15 years.
- Digital records will be encrypted, password protected and kept in a secure server setting.

15.6 Protocol Deviations

Protocol deviations will be reported to the IRB and the Principal Investigator (Dr. Netchiporouk).

16 Publication Policy

Publication will occur expeditiously with full, de-identified data made available.

17. References

1. Organization WH. WHO Coronavirus (COVID-19) Dashboard. Updated April 15 2021. Accessed April 15 2021,
2. Dexamethasone in Hospitalized Patients with Covid-19 — Preliminary Report. *New England Journal of Medicine*. 2020;doi:10.1056/NEJMoa2021436
3. Pan H, Peto R, Karim QA, et al. Repurposed antiviral drugs for COVID-19 –interim WHO SOLIDARITY trial results. *medRxiv*. 2020:2020.10.15.20209817. doi:10.1101/2020.10.15.20209817
4. Beigel JH, Tomashek KM, Dodd LE, et al. Remdesivir for the Treatment of Covid-19 — Final Report. *New England Journal of Medicine*. 2020;383(19):1813-1826. doi:10.1056/NEJMoa2007764
5. Ritchie AI, Singanayagam A. Immunosuppression for hyperinflammation in COVID-19: a double-edged sword? *Lancet*. Apr 4 2020;395(10230):1111. doi:10.1016/S0140-6736(20)30691-7
6. Shi Y, Wang Y, Shao C, et al. COVID-19 infection: the perspectives on immune responses. *Cell Death Differ*. May 2020;27(5):1451-1454. doi:10.1038/s41418-020-0530-3
7. Zhang L, Yan X, Fan Q, et al. D-dimer levels on admission to predict in-hospital mortality in patients with Covid-19. *J Thromb Haemost*. Apr 19 2020;doi:10.1111/jth.14859
8. Ackermann M, Verleden SE, Kuehnel M, et al. Pulmonary Vascular Endothelialitis, Thrombosis, and Angiogenesis in Covid-19. *New England Journal of Medicine*. 2020/07/09 2020;383(2):120-128. doi:10.1056/NEJMoa2015432
9. Logue JK, Franko NM, McCulloch DJ, et al. Sequelae in Adults at 6 Months After COVID-19 Infection. *JAMA Network Open*. 2021;4(2):e210830-e210830. doi:10.1001/jamanetworkopen.2021.0830
10. Chopra V, Flanders SA, O'Malley M, Malani AN, Prescott HC. Sixty-Day Outcomes Among Patients Hospitalized With COVID-19. *Annals of Internal Medicine*. 2020;doi:10.7326/M20-5661
11. Garrigues E, Janvier P, Kherabi Y, et al. Post-discharge persistent symptoms and health-related quality of life after hospitalization for COVID-19. *The Journal of infection*. 2020;81(6):e4-e6. doi:10.1016/j.jinf.2020.08.029
12. Halpin SJ, McIvor C, Whyatt G, et al. Postdischarge symptoms and rehabilitation needs in survivors of COVID-19 infection: A cross-sectional evaluation. *J Med Virol*. Feb 2021;93(2):1013-1022. doi:10.1002/jmv.26368
13. Shi H, Han X, Jiang N, et al. Radiological findings from 81 patients with COVID-19 pneumonia in Wuhan, China: a descriptive study. *Lancet Infect Dis*. Apr 2020;20(4):425-434. doi:10.1016/S1473-3099(20)30086-4
14. Pan F, Ye T, Sun P, et al. Time Course of Lung Changes On Chest CT During Recovery From 2019 Novel Coronavirus (COVID-19) Pneumonia. *Radiology*. Feb 13 2020:200370. doi:10.1148/radiol.2020200370
15. Wang Y, Dong C, Hu Y, et al. Temporal Changes of CT Findings in 90 Patients with COVID-19 Pneumonia: A Longitudinal Study. *Radiology*. Mar 19 2020:200843. doi:10.1148/radiol.2020200843

16. Zhang P, Li J, Liu H, et al. Long-term bone and lung consequences associated with hospital-acquired severe acute respiratory syndrome: a 15-year follow-up from a prospective cohort study. *Bone Res*. 2020;8:8. doi:10.1038/s41413-020-0084-5
17. Das KM, Lee EY, Singh R, et al. Follow-up chest radiographic findings in patients with MERS-CoV after recovery. *Indian J Radiol Imaging*. Jul-Sep 2017;27(3):342-349. doi:10.4103/ijri.IJRI_469_16
18. Polack FP, Thomas SJ, Kitchin N, et al. Safety and Efficacy of the BNT162b2 mRNA Covid-19 Vaccine. *New England Journal of Medicine*. 2020;383(27):2603-2615. doi:10.1056/NEJMoa2034577
19. Baden LR, El Sahly HM, Essink B, et al. Efficacy and Safety of the mRNA-1273 SARS-CoV-2 Vaccine. *New England Journal of Medicine*. 2020;384(5):403-416. doi:10.1056/NEJMoa2035389
20. Abdool Karim SS, de Oliveira T. New SARS-CoV-2 Variants — Clinical, Public Health, and Vaccine Implications. *New England Journal of Medicine*. 2021;doi:10.1056/NEJMc2100362
21. Madhi SA, Baillie V, Cutland CL, et al. Efficacy of the ChAdOx1 nCoV-19 Covid-19 Vaccine against the B.1.351 Variant. *New England Journal of Medicine*. 2021;doi:10.1056/NEJMoa2102214
22. Chen CY, Wang FL, Lin CC. Chronic hydroxychloroquine use associated with QT prolongation and refractory ventricular arrhythmia. *Clin Toxicol (Phila)*. 2006;44(2):173-5. doi:10.1080/15563650500514558
23. Hancox JC, Hasnain M, Vieweg WVR, Crouse ELB, Baranchuk A. Azithromycin, cardiovascular risks, QTc interval prolongation, torsade de pointes, and regulatory issues: A narrative review based on the study of case reports. *Ther Adv Infect Dis*. 2013;1(5):155-165. doi:10.1177/2049936113501816
24. Lu ZK, Yuan J, Li M, et al. Cardiac risks associated with antibiotics: azithromycin and levofloxacin. *Expert Opin Drug Saf*. 2015;14(2):295-303. doi:10.1517/14740338.2015.989210
25. Muir AJ, Arora S, Everson G, et al. A randomized phase 2b study of peginterferon lambda-1a for the treatment of chronic HCV infection. *J Hepatol*. Dec 2014;61(6):1238-46. doi:10.1016/j.jhep.2014.07.022
26. Taniguchi T, Takaoka A. The interferon-alpha/beta system in antiviral responses: a multimodal machinery of gene regulation by the IRF family of transcription factors. *Curr Opin Immunol*. Feb 2002;14(1):111-6. doi:10.1016/s0952-7915(01)00305-3
27. Salama C, Han J, Yau L, et al. Tocilizumab in Patients Hospitalized with Covid-19 Pneumonia. *New England Journal of Medicine*. 2021/01/07 2020;384(1):20-30. doi:10.1056/NEJMoa2030340
28. Lescure F-X, Honda H, Fowler RA, et al. Sarilumab in patients admitted to hospital with severe or critical COVID-19: a randomised, double-blind, placebo-controlled, phase 3 trial. *The Lancet Respiratory Medicine*. doi:10.1016/S2213-2600(21)00099-0
29. Kalil AC, Patterson TF, Mehta AK, et al. Baricitinib plus Remdesivir for Hospitalized Adults with Covid-19. *New England Journal of Medicine*. 2021/03/04 2020;384(9):795-807. doi:10.1056/NEJMoa2031994
30. Feld JJ, Kandel C, Biondi MJ, et al. Peginterferon lambda for the treatment of outpatients with COVID-19: a phase 2, placebo-controlled randomised trial. *The Lancet Respiratory Medicine*. doi:10.1016/S2213-2600(20)30566-X

31. Hung IF-N, Lung K-C, Tso EY-K, et al. Triple combination of interferon beta-1b, lopinavir–ritonavir, and ribavirin in the treatment of patients admitted to hospital with COVID-19: an open-label, randomised, phase 2 trial. *The Lancet*. 2020;395(10238):1695-1704. doi:10.1016/S0140-6736(20)31042-4
32. Zhao M. Cytokine storm and immunomodulatory therapy in COVID-19: Role of chloroquine and anti-IL-6 monoclonal antibodies. *Int J Antimicrob Agents*. Apr 16 2020:105982. doi:10.1016/j.ijantimicag.2020.105982
33. Wysham NG, Sullivan DR, Allada G. An opportunistic infection associated with ruxolitinib, a novel janus kinase 1,2 inhibitor. *Chest*. May 2013;143(5):1478-1479. doi:10.1378/chest.12-1604
34. Harigai M. Growing evidence of the safety of JAK inhibitors in patients with rheumatoid arthritis. *Rheumatology (Oxford)*. Feb 1 2019;58(Suppl 1):i34-i42. doi:10.1093/rheumatology/key287
35. https://www.gene.com/media/press-releases/14747/2018-09-28/fda-approves-genentechs-xolair-omalizuma
36. Kahler KC, Blome C, Forschner A, et al. The outweigh of toxicity versus risk of recurrence for adjuvant interferon therapy: a survey in German melanoma patients and their treating physicians. *Oncotarget*. May 25 2018;9(40):26217-26225. doi:10.18632/oncotarget.25439
37. Kalil AC. Treating COVID-19-Off-Label Drug Use, Compassionate Use, and Randomized Clinical Trials During Pandemics. *JAMA*. Mar 24 2020;doi:10.1001/jama.2020.4742
38. Accessed March 26, 2020. https://health-infobase.canada.ca/datalab/copd-blog.html
39. Evans J, Chen Y, Camp PG, Bowie DM, McRae L. Estimating the prevalence of COPD in Canada: Reported diagnosis versus measured airflow obstruction. *Health Rep*. Mar 2014;25(3):3-11.
40. Johnston SL. Asthma and COVID-19: is asthma a risk factor for severe outcomes? *Allergy*. May 2 2020;doi:10.1111/all.14348
41. Novel Coronavirus Pneumonia Emergency Response Epidemiology T. [The epidemiological characteristics of an outbreak of 2019 novel coronavirus diseases (COVID-19) in China]. *Zhonghua Liu Xing Bing Xue Za Zhi*. Feb 17 2020;41(2):145-151. doi:10.3760/cma.j.issn.0254-6450.2020.02.003
42. Wang Y, Wang Y, Chen Y, Qin Q. Unique epidemiological and clinical features of the emerging 2019 novel coronavirus pneumonia (COVID-19) implicate special control measures. *Journal of Medical Virology*. 2020/03/05 2020;n/a(n/a)doi:10.1002/jmv.25748
43. Mehra MR, Desai SS, Kuy S, Henry TD, Patel AN. Cardiovascular Disease, Drug Therapy, and Mortality in Covid-19. *N Engl J Med*. May 1 2020;doi:10.1056/NEJMoa2007621
44. Abrams EM, Geert WJ, Yang CL. Asthma and COVID-19. *CMAJ*. Apr 24 2020;doi:10.1503/cmaj.200617
45. Abrams EM, Szefler SJ. Managing Asthma during COVID-19: An Example for Other Chronic Conditions in Children and Adolescents. *J Pediatr*. Apr 21 2020;doi:10.1016/j.jpeds.2020.04.049
46. Menzella F, Ghidoni G, Galeone C, Capobelli S, Scelfo C, Facciolongo NC. Immunological Aspects Related to Viral Infections in Severe Asthma and the Role of Omalizumab. *Biomedicines*. Mar 30 2021;9(4)doi:10.3390/biomedicines9040348

47. Inc. NPC. Product Monograph - XOLAIR® (Omalizumab). Accessed March 22, 2020. https://www.novartis.ca/sites/www.novartis.ca/files/xolair_scrip_e.pdf
48. Maltby S, Gibson PG, Powell H, McDonald VM. Omalizumab Treatment Response in a Population With Severe Allergic Asthma and Overlapping COPD. *Chest*. Jan 2017;151(1):78-89. doi:10.1016/j.chest.2016.09.035
49. Gibson PG, Reddel H, McDonald VM, et al. Effectiveness and response predictors of omalizumab in a severe allergic asthma population with a high prevalence of comorbidities: the Australian Xolair Registry. *Intern Med J*. Sep 2016;46(9):1054-62. doi:10.1111/imj.13166
50. Tomomatsu K, Oguma T, Baba T, et al. Effectiveness and Safety of Omalizumab in Patients with Allergic Bronchopulmonary Aspergillosis Complicated by Chronic Bacterial Infection in the Airways. *Int Arch Allergy Immunol*. May 8 2020:1-8. doi:10.1159/000507216
51. Kantor DB, McDonald MC, Stenquist N, et al. Omalizumab Is Associated with Reduced Acute Severity of Rhinovirus-triggered Asthma Exacerbation. *Am J Respir Crit Care Med*. 2016;194(12):1552-1555. doi:10.1164/rccm.201606-1145LE
52. Yalcin AD, Yalcin AN. Future perspective: biologic agents in patients with severe COVID-19. *Immunopharmacol Immunotoxicol*. Feb 2021;43(1):1-7. doi:10.1080/08923973.2020.1818770
53. Netchiporouk E, Nguyen CH, Thuraisingham T, Jafarian F, Maurer M, Ben-Shoshan M. Management of pediatric chronic spontaneous and physical urticaria patients with omalizumab: case series. *Pediatr Allergy Immunol*. Sep 2015;26(6):585-8. doi:10.1111/pai.12407
54. Sundstrom JB, Hair GA, Ansari AA, et al. IgE-FcepsilonRI interactions determine HIV coreceptor usage and susceptibility to infection during ontogeny of mast cells. *J Immunol*. 2009/05// 2009;182(10):6401-6409. doi:10.4049/jimmunol.0801481
55. Khetsuriani N, Kazerouni NN, Erdman DD, et al. Prevalence of viral respiratory tract infections in children with asthma. *Journal of Allergy and Clinical Immunology*. 2007/02/01/ 2007;119(2):314-321. doi:https://doi.org/10.1016/j.jaci.2006.08.041
56. Arden KE, Chang AB, Lambert SB, Nissen MD, Sloots TP, Mackay IM. Newly identified respiratory viruses in children with asthma exacerbation not requiring admission to hospital. *Journal of medical virology*. 2010;82(8):1458-1461.
57. Teach SJ, Gill MA, Togias A, et al. Preseasonal treatment with either omalizumab or an inhaled corticosteroid boost to prevent fall asthma exacerbations. *J Allergy Clin Immunol*. Dec 2015;136(6):1476-1485. doi:10.1016/j.jaci.2015.09.008
58. Esquivel A, Busse WW, Calatroni A, et al. Effects of Omalizumab on Rhinovirus Infections, Illnesses, and Exacerbations of Asthma. *Am J Respir Crit Care Med*. Oct 15 2017;196(8):985-992. doi:10.1164/rccm.201701-0120OC
59. Gill MA, Liu AH, Calatroni A, et al. Enhanced plasmacytoid dendritic cell antiviral responses after omalizumab. *J Allergy Clin Immunol*. May 2018;141(5):1735-1743.e9. doi:10.1016/j.jaci.2017.07.035
60. Gill MA, Bajwa G, George TA, et al. Counterregulation between the FcepsilonRI pathway and antiviral responses in human plasmacytoid dendritic cells. *J Immunol*. 2010;184(11):5999-6006. doi:10.4049/jimmunol.0901194
61. Pernet E, Downey J, Vinh DC, Powell WS, Divangahi M. Leukotriene B4-type I interferon axis regulates macrophage-mediated disease tolerance to influenza infection. *Nat Microbiol*. Aug 2019;4(8):1389-1400. doi:10.1038/s41564-019-0444-3

62. Huang YC, Leyko B, Frieri M. Effects of omalizumab and budesonide on markers of inflammation in human bronchial epithelial cells. *Ann Allergy Asthma Immunol*. Nov 2005;95(5):443-51. doi:10.1016/S1081-1206(10)61170-2
63. Wang T, Hou W, Fu Z. Preventative effect of OMZ-SPT on lipopolysaccharide-induced acute lung injury and inflammation via nuclear factor-kappa B signaling in mice. *Biochem Biophys Res Commun*. Apr 1 2017;485(2):284-289. doi:10.1016/j.bbrc.2017.02.090
64. Tedeschi A, Kolkhir P, Asero R, et al. Chronic urticaria and coagulation: pathophysiological and clinical aspects. *Allergy*. Jun 2014;69(6):683-91. doi:10.1111/all.12389
65. Cugno M, Asero R, Tedeschi A, Lazzari R, Marzano AV. Inflammation and coagulation in urticaria and angioedema. *Curr Vasc Pharmacol*. Sep 2012;10(5):653-8. doi:10.2174/157016112801784558
66. Farres MN, Refaat M, Melek NA, Ahmed EE, Shamseldine MG, Arafa NA. Activation of coagulation in chronic urticaria in relation to disease severity and activity. *Allergol Immunopathol (Madr)*. Mar-Apr 2015;43(2):162-7. doi:10.1016/j.aller.2014.04.002
67. Grzanka R, Damasiewicz-Bodzek A, Kasperska-Zajac A. Interplay between acute phase response and coagulation/fibrinolysis in chronic spontaneous urticaria. *Allergy Asthma Clin Immunol*. 2018;14:27. doi:10.1186/s13223-018-0255-8
68. Kim JA, Kim S, Kim JE, et al. Activation of the Intrinsic Coagulation Pathway in Patients With Chronic Urticaria. *Allergy Asthma Immunol Res*. Sep 2015;7(5):476-82. doi:10.4168/aair.2015.7.5.476
69. Takeda T, Sakurai Y, Takahagi S, et al. Increase of coagulation potential in chronic spontaneous urticaria. *Allergy*. Mar 2011;66(3):428-33. doi:10.1111/j.1398-9995.2010.02506.x
70. Wang D, Tang H, Shen Y, Wang F, Lin J, Xu J. Activation of the Blood Coagulation System in Patients with Chronic Spontaneous Urticaria. *Clin Lab*. 2015;61(9):1283-8. doi:10.7754/clin.lab.2015.150226
71. Yanase Y, Takahagi S, Hide M. Chronic spontaneous urticaria and the extrinsic coagulation system. *Allergol Int*. Apr 2018;67(2):191-194. doi:10.1016/j.alit.2017.09.003
72. Zhu H, Liang B, Li R, et al. Activation of coagulation, anti-coagulation, fibrinolysis and the complement system in patients with urticaria. *Asian Pac J Allergy Immunol*. Mar 2013;31(1):43-50.
73. Asero R. Serial D-dimer plasma levels in a patient with chronic spontaneous urticaria developing resistance to omalizumab. *Clin Exp Dermatol*. Aug 2017;42(6):667-669. doi:10.1111/ced.13181
74. Asero R, Marzano AV, Ferrucci S, Cugno M. Elevated baseline D-dimer plasma levels are associated with a prompt response to omalizumab in patients with severe CSU. *J Allergy Clin Immunol Pract*. Nov - Dec 2017;5(6):1740-1742. doi:10.1016/j.jaip.2017.07.009
75. Asero R, Marzano AV, Ferrucci S, Cugno M. D-Dimer Plasma Levels Parallel the Clinical Response to Omalizumab in Patients with Severe Chronic Spontaneous Urticaria. *Int Arch Allergy Immunol*. 2017;172(1):40-44. doi:10.1159/000453453
76. Cugno M, Genovese G, Ferrucci S, Casazza G, Asero R, Marzano AV. IgE and D-dimer baseline levels are higher in responders than nonresponders to omalizumab in chronic spontaneous urticaria. *Br J Dermatol*. Sep 2018;179(3):776-777. doi:10.1111/bjd.16593

77. Yalcin AD, Celik B, Gumuslu S. D-dimer levels decreased in severe allergic asthma and chronic urticaria patients with the omalizumab treatment. *Expert Opin Biol Ther*. Mar 2014;14(3):283-6. doi:10.1517/14712598.2014.875525
78. Yalcin AD, Cilli A, Bisgin A, Strauss LG, Herth F. Omalizumab is effective in treating severe asthma in patients with severe cardiovascular complications and its effects on sCD200, d-dimer, CXCL8, 25-hydroxyvitamin D and IL-1beta levels. *Expert Opin Biol Ther*. Sep 2013;13(9):1335-41. doi:10.1517/14712598.2013.819338
79. de Montjoye L, Darrigade AS, Gimenez-Arnau A, Herman A, Dumoutier L, Baeck M. Correlations between disease activity, autoimmunity and biological parameters in patients with chronic spontaneous urticaria. *Eur Ann Allergy Clin Immunol*. Jan 20 2020;doi:10.23822/EurAnnACI.1764-1489.132
80. Galvan Casas C, Catala A, Carretero Hernandez G, et al. Classification of the cutaneous manifestations of COVID-19: a rapid prospective nationwide consensus study in Spain with 375 cases. *Br J Dermatol*. Apr 29 2020;doi:10.1111/bjd.19163
81. Ghazal S, Litvinov IV, Aljahani N, Jfri A, Netchiporouk E. Cutaneous Manifestations of Coronavirus Disease 2019 (COVID-19) Infection-What Do We Know So Far? *J Cutan Med Surg*. Jul/Aug 2020;24(4):416-417. doi:10.1177/1203475420928375
82. Adachi M, Kozawa M, Yoshisue H, et al. Real-world safety and efficacy of omalizumab in patients with severe allergic asthma: A long-term post-marketing study in Japan. *Respir Med*. Aug 2018;141:56-63. doi:10.1016/j.rmed.2018.06.021
83. Kirchnerová OR, Valena T, Novosad J, Teřl M, Czech eXpeRience Study G. Real-world effectiveness and safety of omalizumab in patients with uncontrolled severe allergic asthma from the Czech Republic. *Postepy Dermatol Alergol*. 2019;36(1):34-43. doi:10.5114/ada.2018.76606
84. Braunstahl GJ, Chen CW, Maykut R, Georgiou P, Peachey G, Bruce J. The eXpeRience registry: the 'real-world' effectiveness of omalizumab in allergic asthma. *Respir Med*. Aug 2013;107(8):1141-51. doi:10.1016/j.rmed.2013.04.017
85. Reddy VN, Kasahara E, Hiraoka M, Lin LR, Ho YS. Effects of variation in superoxide dismutases (SOD) on oxidative stress and apoptosis in lens epithelium. *Exp Eye Res*. Dec 2004;79(6):859-68. doi:10.1016/j.exer.2004.04.005
86. Baker DL, Nakamura GR, Lowman HB, Fischer SK. Evaluation of IgE Antibodies to Omalizumab (Xolair(R)) and Their Potential Correlation to Anaphylaxis. *AAPS J*. Jan 2016;18(1):115-23. doi:10.1208/s12248-015-9821-x
87. Lieberman PL, Jones I, Rajwanshi R, Rosen K, Umetsu DT. Anaphylaxis associated with omalizumab administration: Risk factors and patient characteristics. *J Allergy Clin Immunol*. Dec 2017;140(6):1734-1736 e4. doi:10.1016/j.jaci.2017.07.013
88. Kim HL, Leigh R, Becker A. Omalizumab: Practical considerations regarding the risk of anaphylaxis. *Allergy, asthma, and clinical immunology : official journal of the Canadian Society of Allergy and Clinical Immunology*. 2010;6(1):32-32. doi:10.1186/1710-1492-6-32
89. Mandel VD, Guanti MB, Liberati S, Demonte A, Pellacani G, Pepe P. Omalizumab in Chronic Spontaneous Urticaria Refractory to Conventional Therapy: An Italian Retrospective Clinical Analysis with Suggestions for Long-Term Maintenance Strategies. *Dermatology and Therapy*. 2018/06/01 2018;8(2):291-301. doi:10.1007/s13555-018-0240-7

90. Wechsler ME, Wong DA, Miller MK, Lawrence-Miyasaki L. Churg-strauss syndrome in patients treated with omalizumab. *Chest*. 2009/08// 2009;136(2):507-518. doi:10.1378/chest.08-2990
91. Iribarren C, Rahmaoui A, Long AA, et al. Cardiovascular and cerebrovascular events among patients receiving omalizumab: Results from EXCELS, a prospective cohort study in moderate to severe asthma. *Journal of Allergy and Clinical Immunology*. 2017;139(5):1489-1495.e5. doi:10.1016/j.jaci.2016.07.038
92. Iribarren C, Rothman KJ, Bradley MS, Carrigan G, Eisner MD, Chen H. Cardiovascular and cerebrovascular events among patients receiving omalizumab: Pooled analysis of patient-level data from 25 randomized, double-blind, placebo-controlled clinical trials. *Journal of Allergy and Clinical Immunology*. 2017;139(5):1678-1680. doi:10.1016/j.jaci.2016.12.953
93. Busse W, Buhl R, Fernandez Vidaurre C, et al. Omalizumab and the risk of malignancy: Results from a pooled analysis. *Journal of Allergy and Clinical Immunology*. 2012;129(4):983-989.e6. doi:10.1016/j.jaci.2012.01.033
94. Long A, Rahmaoui A, Rothman KJ, et al. Incidence of malignancy in patients with moderate-to-severe asthma treated with or without omalizumab. *J Allergy Clin Immunol*. Sep 2014;134(3):560-567.e4. doi:10.1016/j.jaci.2014.02.007
95. Power SP, Moloney F, Twomey M, James K, O'Connor OJ, Maher MM. Computed tomography and patient risk: Facts, perceptions and uncertainties. *World J Radiol*. 2016;8(12):902-915. doi:10.4329/wjr.v8.i12.902
96. Sakane H, Ishida M, Shi L, et al. Biological Effects of Low-Dose Chest CT on Chromosomal DNA. *Radiology*. 2020/05/01 2020;295(2):439-445. doi:10.1148/radiol.2020190389
97. Morais-Almeida M, Aguiar R, Martin B, et al. COVID-19, asthma, and biological therapies: What we need to know. *World Allergy Organ J*. 2020;13(5):100126-100126. doi:10.1016/j.waojou.2020.100126
98. Ghazawi FM, Lim M, Dutz JP, Kirchhof MG. Infection risk of dermatologic therapeutics during the COVID-19 pandemic: an evidence-based recalibration. *Int J Dermatol*. 2020/09// 2020;59(9):1043-1056. doi:10.1111/ijd.15028
99. Zhao Y-m, Shang Y-m, Song W-b, et al. Follow-up study of the pulmonary function and related physiological characteristics of COVID-19 survivors three months after recovery. *EClinicalMedicine*. 2020;25doi:10.1016/j.eclinm.2020.100463
100. Abdelmaksoud A, Goldust M, Vestita M. Omalizumab and COVID-19 treatment: Could it help? *Dermatol Ther*. Jun 8 2020:e13792. doi:10.1111/dth.13792
101. Cao B, Wang Y, Wen D, et al. A Trial of Lopinavir–Ritonavir in Adults Hospitalized with Severe Covid-19. *New England Journal of Medicine*. 2020;doi:10.1056/NEJMoa2001282
102. Organization WH. WHO R&D Blueprint: novel Coronavirus - Outline of designs for experimental vaccines and therapeutics Accessed March 24, 2020. https://www.who.int/blueprint/15-01-2020-nfr-bp-wg-clinical-trials-ncov.pdf?ua=1
103. Chang YC, Yu CJ, Chang SC, et al. Pulmonary sequelae in convalescent patients after severe acute respiratory syndrome: evaluation with thin-section CT. *Radiology*. Sep 2005;236(3):1067-75. doi:10.1148/radiol.2363040958
104. Namazy JA, Blais L, Andrews EB, et al. Pregnancy outcomes in the omalizumab pregnancy registry and a disease-matched comparator cohort. *J Allergy Clin Immunol*. Feb 2020;145(2):528-536.e1. doi:10.1016/j.jaci.2019.05.019

105. International Council for Harmonisation of Technical Requirements for Pharmaceuticals for Human Use (ICH). Safety Guidelines. Accessed October 29, 2020. https://www.ich.org/page/safety-guidelines
106. Novartis Pharmaceuticals Canada Inc. Product Monograph Xolair (Omalizumab). September 26 2017 2017;
107. Azer SA. COVID-19: pathophysiology, diagnosis, complications and investigational therapeutics. *New Microbes New Infect*. Sep 2020;37:100738. doi:10.1016/j.nmni.2020.100738
